# Contribution to the Diversity of the Genus *Sarcophaga* (Diptera: Sarcophagidae): Checklist, Species Distribution, and New Records for Greece

**DOI:** 10.3390/insects16040359

**Published:** 2025-03-31

**Authors:** Savvas Zafeiriou, Gabriella Dimitra Rakopoulou, Georgios Agapakis, Theodora Petanidou, Sotiris Alexiou

**Affiliations:** 1 Laboratory of Biogeography and Ecology, Department of Geography, University of the Aegean, University Hill, GR 81100 Mytilene, Greece; t.petanidou@aegean.gr; 2 Entomology and Nematology Department, University of Florida, Gainesville, FL 32611, USA; gabriella.rakopoulou1998@gmail.com; 3 Department of Biotechnology, Agricultural University of Athens, GR 11855 Athens, Greece; gagapakes@gmail.com; 4 Korinthian Museum of Natural History, Isthmion 201, GR 20100 Korinthos, Greece; info@korinthianmuseumnaturalhistory.com

**Keywords:** flesh flies, forensic entomology, taxonomy, biodiversity, Greece

## Abstract

The current study presents new records and an updated checklist of the genus *Sarcophaga* (Diptera: Sarcophagidae) for Greece. The records were compiled from published literature, identification of newly collected material obtained through passive (trapping) and active (hand collecting, net sweeping) sampling conducted between 2018 and 2024, and the examination of material from two museum collections: Entomological Collection of the Goulandris Natural History Museum (1983–1987) and the Melissotheque of the Aegean (2004–2024). After the examination of 927 male specimens from 108 localities and the synthesis of the available literature, a total of 72 species distributed across 18 subgenera were documented. Among these, *Sarcophaga ferox* (Villeneuve, 1908) and *Sarcophaga anaces* (Walker, 1849) are newly records for Greece, while *Sarcophaga beckiana* (Lehrer, 1996) represents the first documented occurrence of the species in Europe. The findings represent the most contemporary contribution to the understanding of the species diversity and distribution of the genus *Sarcophaga* in Greece.

## Simple Summary

The flesh flies (Diptera: Sarcophagidae) hold scientific value in forensic science, have implications for veterinary research, and contribute to ecosystem dynamics. Despite their importance, their diversity in Greece remains largely uncharted. The present study delivers the first checklist of *Sarcophaga* species in the country, based on literature records, museum collections, and collected specimens from 2018 to 2024. A total of 72 species are confirmed, including two newly recorded in Greece [*Sarcophaga ferox* (Villeneuve, 1908), *Sarcophaga anaces* Walker, 1849] and one documented for the first time in Europe [*Sarcophaga beckiana* (Lehrer, 1996)]. By mapping their distribution and species composition, this research provides important data for regional forensic investigations, biodiversity studies, and ecological monitoring. These findings lay the groundwork for future research and emphasize the ongoing need for targeted collections and taxonomic studies to uncover the full diversity of *Sarcophaga* species in Greece.

## 1. Introduction

The family Sarcophagidae, commonly known as flesh flies, originates from the Ancient Greek terms [“*sarx*”—(“flesh”)] and [“*phagein*”—(“to eat”)] [1], reflecting the necrophagous behavior exhibited by a substantial number of species within the taxon [2]. Most species within the family adopt an ovoviviparous reproductive strategy, depositing first-instar larvae directly onto suitable substrates, a process known as larviposition [3]. Flesh fly species vary widely in size, ranging from 2 to 22 mm in total body length [4]. Sarcophagids are distributed across all zoogeographical regions of the world and encompass a significant number of species that are ubiquitous and closely associated with human environments [2,5]. The family Sarcophagidae constitutes a megadiverse taxonomic group, comprising approximately 3000 species described globally [6]. The taxon is systematically divided into three subfamilies: Miltogramminae, Paramacronychiinae, and Sarcophaginae [7,8,9]. Of these, Sarcophaginae constitutes the largest subfamily, encompassing over 2200 species distributed across 51 genera [5].

Among the members of this subfamily, the highly speciose genus *Sarcophaga* Meigen (Sarcophagidae, Sarcophaginae) represents the largest radiation within Sarcophagidae [10] and also one of the largest genera of Diptera [11]. The genus is monophyletic [10] and accounts for nearly one-third of all described sarcophagid species [5,6], comprising approximately 890 species systematically arranged into 169 subgenera [10]. *Sarcophaga* species are widespread, showing the greatest diversity in the Holarctic, Oriental, and Afrotropical regions [12].

*Sarcophaga* spp. exhibit uniform coloration patterns, typically characterized by a gray thorax with three distinct blackish vittae and a tessellated or checkerboard-like abdomen, which alters in appearance depending on light incidence [5,12]. The external morphology of adult *Sarcophaga* spp. flies is highly homogeneous, rendering species-level identification possible only through the examination of the terminalia, particularly the complex structural morphology of the distiphallus in males, or, when documented, in females [10].

Adults feed on various resources, including nectar, sap, pollen, fruit juices, and honeydew and are very often visiting decomposing organic matter, such as excrement and carrion [4,13]. Numerous species within this group are of significant medical and veterinary importance, serving as facultative myiasis agents and mechanical vectors for a diverse range of pathogenic microorganisms [14,15]. Others exhibit predatory and/or parasitic behavior targeting various invertebrate hosts as part of their ecological diversity [16]. Additionally, the taxon comprises a substantial number of necrophagous species that are frequently encountered in carrion arthropod assemblages, where they contribute significantly to the decomposition of vertebrate cadavers [6]. They are also frequent colonizers of human corpses and, as such, are among the main insect groups when forensic entomology is used in casework [6].

Historically, research on Greek *Sarcophaga* has remained limited to a small number of species descriptions and some rare referrals in a few major, extralimital studies [2,17,18,19,20,21,22]. It was not until the publication of the first dedicated catalog for the family in 1996 [5], when the first pictures appeared concerning the total number of Greek species. At roughly the same time, the notable dipterologist Dalibor Povolný carried out a number of targeted taxonomical and ecological studies concerning the Greek mainland (mostly the areas of Epirus and Macedonia) that resulted in the description of a few more species and the better understanding of the distribution of some *Sarcophaga* spp. in these regions [23,24,25,26,27]. Following the publications of Povolný, no more studies on the genus have been produced for Greece. All subsequent species records are the result of examined museum materials on the subgenus *Heteronychia* [12,28,29,30], including updates on the 1996 catalog [31,32], extralimital checklists incorporating new specimens [33], and non-targeted ecological studies [34,35,36]. As a result of all these studies, there are presently 69 recorded Greek species. However, there is a considerable knowledge gap for the mainland (e.g., Thrace and Peloponnese) and most islands [2,5,26,27,30,31,32,33]. As a consequence, the overarching aim of this research is to consolidate the available data on the genus *Sarcophaga* in Greece through the compilation of a species checklist, which synthesizes previously published records, incorporates data from newly collected flesh fly specimens, and examines materials from two museum collections. The outcomes of this study will provide a provisional basis for advancing systematic studies in Greece and establish a framework for future faunistic, ecological, and forensic research on this taxonomic group.

## 2. Materials and Methods

### 2.1. Methodology

The present study is based on an examination of 927 male dry-pinned specimens collected from various places in Greece between 1984 and 2024. The specimens have been preserved in the following entomological collections: the Entomological Collection of the Goulandris Natural History Museum, Athens, Greece (GNHM); the Korinthian Museum of Natural History Museum, Korinthos, Greece (KNMH); the Melissotheque of the Aegean, Mytilene, Greece [37]; and private collections of the following authors (S.Z., G.D.R., G.A., and S.A.).

Flesh fly specimens were collected using both passive and active sampling techniques to maximize collection efficiency. Passive sampling involved the deployment of baited bottle and pitfall traps, each containing 50 g of beef liver, 50 g of chicken stomach, or ~50–60 g of whole sardine fish, following the methodology outlined by Rakopoulou and Dadour [38]. Active sampling was performed using hand nets and manual collection, enabling the direct capture of adult *Sarcophaga* species. Insects deposited in the Melissotheque of the Aegean were collected from several Greek island and mountain sites using the pantrap and hand-netting protocol [39,40]. For each site, 3 visits were made during the main flowering season which incorporated 10 pantrap triplets. Each triplet consisted of three UV-bright pantraps of yellow, blue, and white colors. Each pantrap was filled with 350 mL of water in which one drop of aroma-free detergent was added and left onsite for 48 h before collection. Hand sampling was carried out for 120 min per site and visit using hand nets, collecting insects observed on flowers along specific walks. Insects collected were brought to the lab for further processing and identification.

In the laboratory, all insect specimens were pinned, enumerated, and sexed. Taxonomic identification to species level was restricted to male flesh fly specimens. This necessitated the dissection and examination of the male aedeagal structures. Terminalia were exposed using entomological tweezers and immersed in a 10% potassium hydroxide (KOH) solution to render the soft tissues translucent. Identifications were conducted using a stereo microscope (Model: BMS 133 Zoom Trino, LED, BMS Microscopes b.v.; Capelle aan den IJssel; the Netherlands) and validated through comparison with specialized taxonomic keys, revisions, descriptions, and authoritative publications [2,25,28,29,41,42,43]. The nomenclature and classification adopted in this study follows the updated versions of the 1996 catalog [31,32] with slight adjustments, based on recent studies on specific subgenera and species [29,30,44].

**Figure 1:**
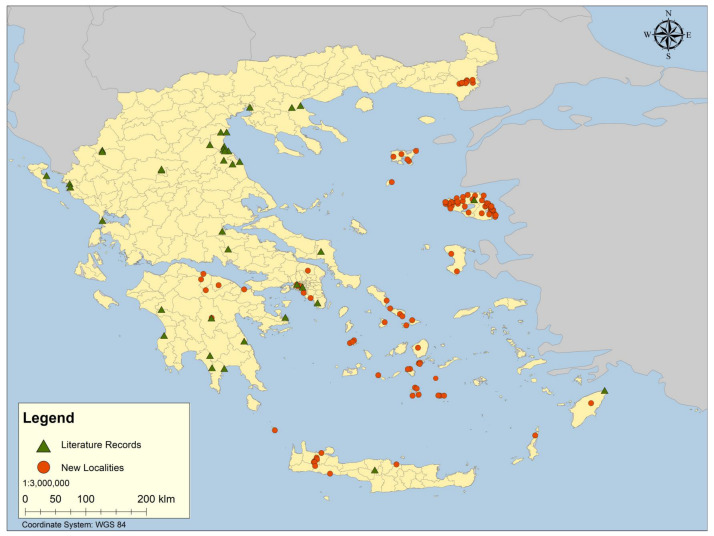
Map depicting all studied localities in Greece, with green triangles denoting literature records and red circles representing localities documented in the present study. (The map was generated using ArcMap 10.2.2.).

Data for European and worldwide distributions for the species are derived from multiple sources [2,5,20,21,22,23,26,27,28,29,30,31,32,33,43,45,46,47,48]. Geographic localities for Greek sampling sites provided in the literature are presented in Table 1, while localities containing newly collected and examined material are provided in Table 2. Literature records and all newly studied localities are presented in Figure 1.

### 2.2. Format of Checklist

The systematic checklist is divided into two distinct parts: The first part contains records of valid species arranged in alphabetical order within their appropriate subgenera. Each species entry starts with its current specific name, the authority, and the year of publication. When material is available for examination, it is listed immediately afterwards. In each case, the regional unit or island from which the specimen originated is underlined and capitalized, followed by the locality of collection, the number and sex of each specimen, and the date of collection. Different entries are separated by semicolons. This is followed by the known Greek distribution of the regional area and, when available, islands, along with the relevant bibliographic records and the used synonyms. Subsequently, the general distribution for each species is given at country level, while a few notable regions are mentioned separately (e.g., Canary Isl. and Madeira). Remarks follow for a number of species, where extra taxonomical, ecological, and biological information is provided. Species recorded in Greece for the first time are marked with a black triangle (^▲^), new records within the country are indicated with an asterisk (*) and doubtful distributional records with a question mark (?).

The second part of the checklist contains the records of the taxa mentioned in the literature for which no safe identification was possible due to the taxonomic perplexity or problems with the examination of the available material (inability to access it or damaged specimens). These species entries are constructed in a shorter but similar format to the first part of the checklist. They are arranged by subgenera in alphabetical order, taxa included in brackets, followed by the known distribution in Greece based on the literature records. Finally, a “Remarks” section is also given, explaining possible identities and the reasons why these specimens were unable to be properly categorized.

## 3. Results

**Figure 2:**
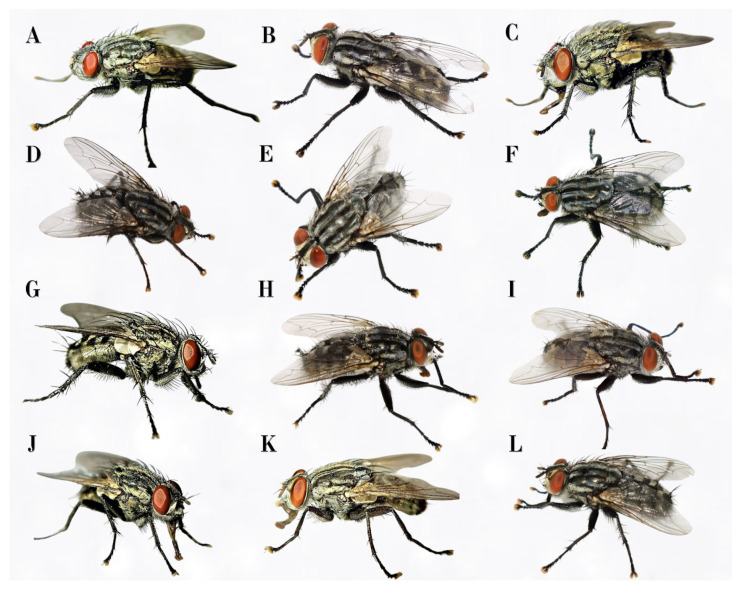
Adult male external morphology of selected *Sarcophaga* species. (**A**): *S. ferox*; (**B**): *S. crassipalpis*; (**C**): *S. thirionae*; (**D**): *S. bellae*; (**E**): *S. argyrostoma*; (**F**): *S. beckiana*; (**G**): *S. boettcheri*; (**H**): *S. lehmanni*; (**I**): *S. africa*; (**J**): *S. minima*; (**K**): *S. tibialis*; (**L**): *S. incisilobata*.

***Sarcophaga* Meigen, 1826**
***Sarcophaga* (*Bercaea*) *africa* (Wiedemann, 1824) (Figure 2I)**

**Material Examined:** ACHAIA*: Potamia, 1♂, 12 July 2018; 1♂, 16 July 2018; 5♂, 25 July 2019; ATTIKI: Agia Marina, 1♂, 3 April 2022; 1♂, 12 November 2022; Agia Varvara, 1♂, 20 December 2022; 1♂, 4 April 2023; Althea Beach, 1♂, 21 June 2022; Agricultural University of Athens, 9♂, 24 May 2021; 5♂, 26 May 2021; 1♂, 30 May 2021; 3♂ 9 June 2021; 4♂, 14 June 2021; 2♂, 22 June 2021; 3♂, 24 June 2021; 15♂, 28 June 2021; 1♂, 3 July 2021; 7♂, 8 July 2021; 2♂, 13 July 2021; 7♂, 16 July 2021; 1♂, 19 July 2021; 4♂, 23 July 2021; 1♂, 30 July 2021; 7♂, 19 August 2021; 1♂, 8 September 2021; 2♂, 13 September 2021; 3♂, 21 September 2021; 10♂, 27 September 2021; 4♂, 7 October 2021; 5♂, 18 October 2021; 1♂, 22 October 2021; 1♂, 27 October 2021; 1♂, 29 October 2021; Diomedes Botanical Garden, 1♂, 4 May 2023; Ellinikon International Airport, 1♂, 13 June 2022; 1♂, 9 April 2023; CHANIA*: Alikianos, 1♂, 2 November 2023; EVROS*: Dadia III, 1♂, 23 August 2012; IRAKLEIA*: Irakleia, 6♂, 12 August 2020; KORINTHIA*: Lechaio, 1♂, 22 April 2022; LESVOS*: Moni Ipsilou II, 1♂, 16 August 2023; Mytilene, 2♂, 8 June 2004; Pirgi Thermis, 1♂, 18 December 2022; 1♂, 21 September 2023; 1♂, 24 October 2023; 1♂, 19 October 2024 Polichnitos, 1♂, 4 October 2022; Vathylimno Waterfalls, 1♂, 14 September 2024; Vigla Pamfilon, 1♂, 5 August 2023; SYROS*: Ermoupoli, 1♂, 11 September 2023.

**Distribution in Greece:** Known as *Sarcophaga cruentata* from the provinces of Ioannina, Preveza, Thesprotia, Pieria, Trikala, and Attiki [23,26,27]. New for Thrace, Peloponnese, North Aegean Isl., Cyclades, and Crete.

**General Distribution:** Afghanistan, Albania, Algeria, Angola, Argentina, Armenia, Australia, Austria, Azerbaijan, Azores, Bahrain, Belarus, Belgium, Benin, Bhutan, Botswana, Brazil, Bulgaria, Burkina Faso, Burundi, Cameroon, Canada, Canary Isl., China, Costa Rica, Croatia, Cuba, Cyprus, Czech Republic, Denmark, Egypt, Eritrea, Ethiopia, France, Gabon, Gambia, Germany, Greece, Gruzia, Hungary, India, Iran, Iraq, Ireland, Israel, Italy, Ivory Coast, Japan, Kazakhstan, Kenya, Kuwait, Kyrgyzstan, Latvia, Lebanon, Lesotho, Liberia, Libya, Lithuania, Luxembourg, Madagascar, Madeira, Mali, Malta, Mauritania, Mauritius, Malaysia, Mexico, Moldova, Mongolia, Morocco, Mozambique, Namibia, Nepal, Netherlands, Nicobar Isl., Niger, Nigeria, North Korea, North Macedonia, Norway, New Caledonia, Pakistan, Paraguay, Poland, Portugal, Réunion, Romania, Russia, Rwanda, Saudi Arabia, Serbia, Seychelles, Sierra Leone, Slovakia, South Africa, South Korea, Spain, Saint Helena, Sudan, Sweden, Switzerland, Syria, Tajikistan, Tanzania, Thailand, Togo, Tunisia, Turkey, Turkmenistan, Ukraine, United Kingdom, USA, Uzbekistan, Vietnam, Yemen, Zaire, Zambia, and Zimbabwe [5,31,32,33,46].

**Remarks:** The species shows hemisynanthropic and culturophilic tendencies, as it is frequently associated with urban environments [2,49]. It has also been reported in laystalls, marshy, sandy, and pond habitats [50]. Larvae are considered primarily coprophagous (coprobiodotic), and when given the choice, females oviposit almost exclusively on feces [2,13,51]. In addition, larvae develop in a large variety of organic substrates, including living acridoid grasshoppers, terrestrial snails, rotten meat, carcasses (both vertebrate and invertebrate), and birds’ nests [2,32,43,52]. However, many of the non-fecal records are provided by non-taxonomists and, in combination with the common name given to the species (“Red-tailed Flesh Fly”), should be accepted with caution, as they may refer to other species with reddish genitalia [2]. The species is of significant medical importance, as it is able to cause myiasis in animals and humans, aurally, dermally, and intestinally in the latter case [2,21,32,43,51]. It is also a known passive vector of bacteria causing dysentery, protozoan cysts, tapeworm oncospheres and nematode eggs [2].

***Sarcophaga* (*Helicophagella*) *agnata* Rondani, 1861**

**Distribution in Greece:** Known from the province of Trikala [26,27].

**General Distribution:** Albania, Austria, Belgium, Bulgaria, Croatia, Czech Republic, Denmark, France, Germany, Greece, Hungary, Italy, Kazakhstan, the Netherlands, Norway, Poland, Romania, Russia, Slovakia, Spain, Sweden, Switzerland, Ukraine, and United Kingdom [2,5,26,27,31,32,33].

**Figure 3:**
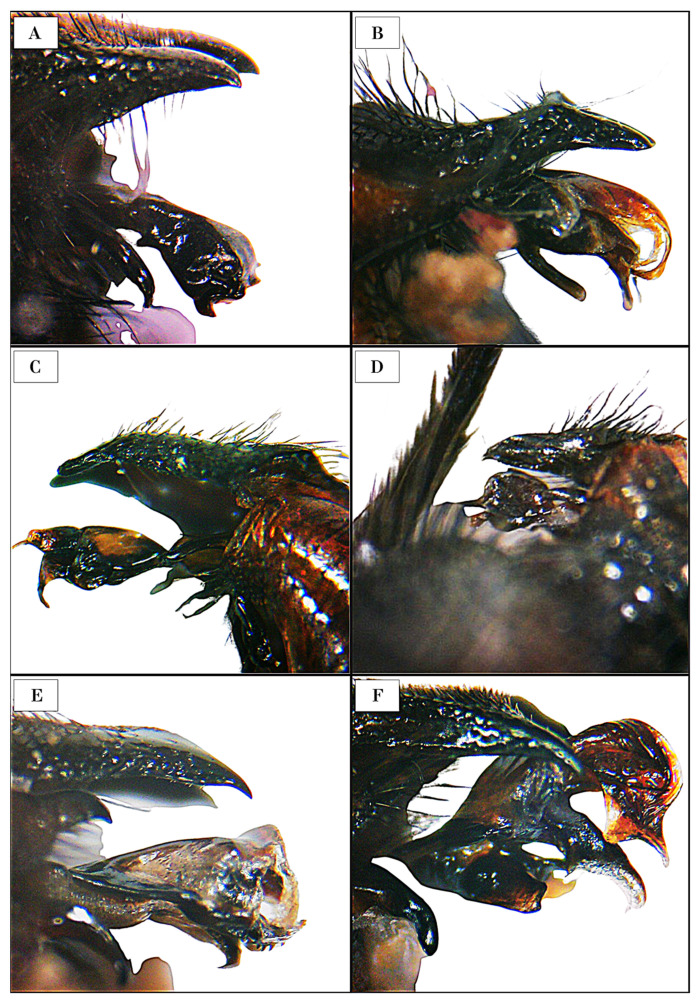
Illustration of dissected male aedeagal structures of selected *Sarcophaga* spp. (**A**): *S. bellae*; (**B**): *S. ferox*; (**C**): *S. pandellei*; (**D**) *S. anaces*; (**E**): *S. czernyi*; (**F**): *S. beckiana*.

***Sarcophaga* (*Helicophagella*) *bellae* (Lehrer, 2000) (** Figure 2 **D and** Figure 3 **A)**

**Material Examined:** LESVOS*: Alyfanta, 1♂, 17 February 2024; Castle of Mytilene, 1♂, 27 March 2024; and Latomeio Eresou, 1♂, 30 April 2011.

**Distribution in Greece:** Known from mainland Greece without specific localities [31,32]. New for North Aegean Isl.

**General Distribution:** Greece, Israel, and Turkey [31,32,52].

**Remarks:** The species, already known from Israel and Turkey, has been reported from Greece in recent catalogs without precise locality data and associated material [31,32]. As such, the abovementioned specimens constitute the first published examined material for *S. bellae* in Greece and verify its presence in the country.

***Sarcophaga* (*Helicophagella*) *crassimargo* Pandellé, 1896**

**Distribution in Greece:** Known from mainland Greece without specific localities [5,31,32,33].

**General Distribution:** Albania, Austria, Azerbaijan, Belgium, Bulgaria, China, Croatia, Czech Republic, Denmark, Finland, France, Germany, Greece, Gruzia, Hungary, Ireland, Italy, Kazakhstan, Kyrgyzstan, Latvia, Lithuania, Macedonia, Moldova, the Netherlands, Norway, Poland, Romania, Russia, Serbia, Slovakia, Spain, Sweden, Switzerland, Turkey, Ukraine, and United Kingdom [5,31,32,33,47].

**Remarks:** The species has been reported in laystalls, marshy, sandy, and pond habitats [50]. Larvae are considered copro-necrophagous [2]. Occasional reports of development in terrestrial snails are probably erroneous [43].

***Sarcophaga* (*Helicophagella*) *hirticrus* Pandellé, 1896**

**Material Examined:** ANAFI*: Vagia, 2♂, 13 May 2013; ATTIKI*: Agricultural University of Athens, 1♂, 24 May 2021; 2♂, 9 June 2021; 2♂, 14 June 2021; 4♂, 24 June 2021; 3♂, 28 June 2021; 2♂, 28 June 2021; 1♂, 8 July 2021; 1♂, 13 July 2021; 3♂, 16 July 2021; 1♂, 30 July 2021; 1♂, 10 August 2021; 1♂, 7 October 2021; 1♂, 27 October 2023; CHANIA*: Alikianos, 1♂, 8 May 2023; 4♂, 26 May 2023; Kefali, 2♂, 28 March 2023; 3♂, 8 May 2023; 1♂, 26 May 2023; Lakkoi, 1♂, 8 May 2023; EVROS *: Dadia VII, 1♂, 23 September 2012; FOLEGANDROS*: Agios Georgios, 1♂, 13 June 2014; IRAKLEIA*: Irakleia, 5♂, 12 August 2020; Livadi, 1♂, 17 May 2014; LESVOS*: Castle of Mytilene, 1♂, 25 April 2023, 1♂, 27 March 2024; Loutropoli Thermis, 1♂, 21 June 2022; LIMNOS*: Moudros I, 1♂, 13 June 2012; Plaka-Panagia, 1♂, 5 April 2012; 1♂, 13 June 2012.

**Distribution in Greece:** Known from the provinces of Ioannina, Pieria, and Trikala [26,27]. New for Thrace, North Aegean Isl., Cyclades, and Crete.

**General Distribution:** Albania, Algeria, Andorra, Austria, Azerbaijan, Belgium, Bulgaria, Canary Islands, Croatia, Czech Republic, France, Germany, Greece, Gruzia, Hungary, Italy, Malta, Norway, Poland, Portugal, Romania, Russia, Serbia, Slovakia, Spain, Sweden, Switzerland, Turkey, Ukraine, and United Kingdom [5,26,27,31,32,33,47].

**Remarks:** The species is considered heliophilic and has been reported from urban environments [2,42,49]. Larvae develop on living terrestrial snails, various insects, and carcasses [2,30,43,51,53].

***Sarcophaga* (*Helicophagella*) *maculata* Meigen, 1835**

**Distribution in Greece:** Known from the province of Pieria [26,27].

**General Distribution:** Algeria, Azores, Canary Is, Egypt, France, Germany, Greece, Israel (?), Italy, Lebanon (?), Morocco, Poland, Saudi Arabia, Spain, Syria (?), Tunisia, and Turkey [5,26,27,31,32,46,47].

**Remarks:** The species is a synanthropic visitor of feces [2]. Larvae have been reported to develop in feces and carcasses (both from vertebrates and invertebrates), but these records need verification due to confusion with other, morphologically similar species [22,42,43]. An old breeding record from a tenebrionid beetle *Pimelia grandis latastei* (Sénac, 1884) is dubious and probably erroneous [42,43,54].

***Sarcophaga* (*Helicophagella*) *melanura* Meigen, 1826**

**Material Examined:** ATTIKI: Agricultural University of Athens, 4♂, 24 May 2021; 1♂, 26 May 2021; 4♂, 30 May 2021; 7♂, 9 June 2021; 4♂, 14 June 2021; 4♂, 22 June 2021; 1♂, 24 June 2021; 5♂, 28 June 2021; 3♂, 8 July 2021; 1♂, 13 July 2021; 3♂, 16 July 2021; 3♂, 19 July 2021; 4♂, 10 August 2021; 1♂, 19 August 2021; 3♂, 18 October 2021; 1♂, 27 October 2021; Ellinikon International Airport, 1♂, 11 March 2023; IRAKLEIA*: Irakleia, 2♂, 12 August 2020; KORINTHIA*: Lechaio, 1♂, 10 June 2023.

**Distribution in Greece:** Known from the provinces of Thesprotia, Preveza, Pieria, Trikala, and Attiki [23,26,27]. New for Peloponnese and Cyclades.

**General Distribution:** Afghanistan, Albania, Algeria, Armenia, Austria, Azerbaijan, Belarus, Belgium, Bulgaria, Canada, Canary Isl., China, Croatia, Cyprus, Czech Republic, Denmark, Egypt, Finland, France, Germany, Greece, Gruzia, Hungary, India, Iran, Iraq, Ireland, Israel, Italy, Japan, Kazakhstan, Kyrgyzstan, Latvia, Lithuania, Luxembourg, Malaysia, Malta, Mauritania, Moldova, Mongolia, Morocco, the Netherlands, North Korea, North Macedonia, Norway, Pakistan, Poland, Portugal, Romania, Russia, Serbia, Slovakia, South Korea, Spain, Sweden, Switzerland, Syria, Taiwan, Tajikistan, Tunisia, Turkey, Turkmenistan, Uzbekistan, Ukraine, United Kingdom, and USA [5,31,32,46,47].

**Remarks:** Hemisynanthropic and culturophilic species which has also been reported in laystalls, marshy, sandy, and pond habitats [2,50]. Larvae develop in feces on vertebrate and invertebrate carcasses, privies, garbage, living terrestrial snails, and grasshoppers and are predators of other saprophagous larvae (Diptera) [2,43,55]. The species is of medical importance, as it is known to cause myiasis in mammals, including hedgehogs (Erinaceidae) and humans, and to transfer pathogenic bacteria and eggs of helminths [2,43,55].

***Sarcophaga* (*Helicophagella*) *novercoides* Böttcher, 1913**

**Material Examined:** AGIOS EFSTRATIOS*: Alonitsi Beach, 1♂, 8 May 2022; ANAFI*: Zoodohos Pigi, 1♂, 12 May 2013; CHANIA*: Kefali, 2♂, 28 March 2023; 1♂, 26 May 2023; Lakkoi, 1♂, 28 March 2023; 1♂, 2 November 2023; Omalos III, 1♂, 10 August 2023; FOLEGANDROS*: Agios Georgios, 1♂, 13 June 2014; IRAKLEIA*: Irakleia, 2♂, 12 August 2020; Livadi, 1♂, 25 April 2013; KORINTHIA*: Kato Trikala, 1♂, 17 April 2019; LESVOS*: Castle of Mytilene, 1♂, 25 April 2023; 2♂, 27 March 2024; Latomeio Eresou, 2♂, 30 Apri 2011; Moria, 1♂, 13 October 2022; Petrified Forest Park “Bali Alonia”, 1♂, 11 February 2021.

**Distribution in Greece:** Known from the provinces of Ioannina, Pieria, Trikala, and Attiki [26,27,34,35]. New for Peloponnese, North Aegean Isl., Cyclades, and Crete.

**General Distribution:** Albania, Algeria, Austria, Bulgaria, Croatia, Cyprus, Egypt, France, Germany, Greece, Hungary, Israel, Italy, Malta, Montenegro, Morocco, Russia, Serbia, Slovakia, Spain, Switzerland, Turkey, and Ukraine [5,31,32,33,46,47].

**Remarks:** It is considered a purely Mediterranean, probably lowland-restricted, species, as determined by Povolný and Verves (1997) [2]. Larvae records as parasitoids of terrestrial snails and insects need verification due to possible confusion with other species of this complex [42].

***Sarcophaga* (*Heteronychia*) *ancilla* Rondani, 1865**

**Distribution in Greece:** Known from the province of Pieria [25,26,27].

**General Distribution:** Armenia, Austria, Azerbaijan, Bulgaria, Croatia, Czech Republic, France, Greece, Gruzia, Hungary, Italy, Romania, Russia, Serbia, Slovakia, Spain, Switzerland, and Ukraine [5,30,31,32,33].

**Remarks:** The species was confused for a long time with the morphologically similar and formerly synonymous *Sarcophaga belanovskyi* [29,30]. As a result, the distributional records of *S. ancilla* are in need of careful revision as the species may in fact be restricted to Western Europe [29,30,33]. Considered, while not differentiated by *S. belanovskyi*, as it is commonly found in the limestone mountains of Greece [2].

***Sarcophaga* (*Heteronychia*) *belanovskyi* (Verves, 1973)**

**Distribution in Greece:** Known from mainland Greece without specific localities [30].

**General Distribution:** Austria, Azerbaijan, Bulgaria, Croatia, Czech Republic, Greece, Gruzia, Hungary, Italy, Romania, Russia, Serbia, and Ukraine [30,32,33].

**Remarks:** Formerly considered a synonym of *Sarcophaga ancilla* and may replace this in parts of its range (see also under *S. ancilla*) [29,30,33].

***Sarcophaga* (*Heteronychia*) *benaci* Böttcher, 1913**

**Distribution in Greece:** Known from the provinces of Ioannina, Thesprotia, Pieria, Arcadia, and Ilia [25,26,27,29,30].

**General Distribution:** Albania, Andorra, Austria, Bulgaria, Czech Republic, Croatia, Germany, Greece, Italy, Malta, Norway, Poland, Romania, Serbia, Slovakia, Spain, Sweden, Switzerland, and Turkey [5,30,31,32,33,47].

**Remarks:** Larvae develop as parasitoids of terrestrial snails in the genera *Chondrina* and *Clausilia* [21,30].

***Sarcophaga* (*Heteronychia*) *boettcheri* Villeneuve, 1912 (Figure 2G)**

**Material Examined:** ATTIKI: Agricultural University of Athens, 2♂, 14 June 2021; 2♂, 8 July 2021; 1♂, 13 July 2021; 1♂, 18 July 2021; 1♂, 22 July 2021; Ellinikon International Airport, 1♂, 11 March 2023; LESVOS*: Pirgi Thermis, 1♂, 15 October 2020.

**Distribution in Greece:** Known as *Sarcophaga boettcheri* and *S. taurica* from the provinces of Pieria, Phthiotis, Attiki (mainland and Poros Isl.), and Cyclades [2,5,22,25,26,27,29,30,33,34,35]. New for North Aegean Isl..

**General Distribution:** Austria, Azerbaijan, Bulgaria, Croatia, Cyprus, Greece, Hungary, Iran, Israel, Palestine, Romania, Serbia, Syria, Turkey, and Ukraine [5,30,31,32,33,47].

**Remarks:** Larvae develop as parasitoids of the terrestrial snail *Theba pisana* (O. F. Müller, 1774) [30,56].

***Sarcophaga* (*Heteronychia*) *chaetoneura* (Brauer & Bergenstamm, 1889)**

**Distribution in Greece:** Known from the province of Thessaloniki [30].

**General Distribution:** Austria, Belarus, Czech Republic, France, Greece, Germany, Hungary, Italy, Latvia, Slovakia, and Ukraine [5,30,31,32].

**Remarks:** The species was confused for a long time with the morphologically similar and formerly synonymous *Sarcophaga dissimilis* Meigen, 1826 [2,5,29].

***Sarcophaga* (*Heteronychia*) *consanguinea* Rondani, 1860**

**Material Examined:** DELOS*: Delos, 1♂, 9 July 2015; LESVOS*: Antissa I, 1♂, 23 April 2011; Castle of Mytilene, 30♂, 27 March 2024; 1♂, 30 April 2024; Mytilene, 2♂, 8 May 2004; Parakoila, 1♂, 9 June 2012; Petalidi Beach, 1♂, 19 September 2022; LIMNOS*: Atsiki, 4♂, 15 May 2012; 2♂, 14 June 2012; Moudros I, 1♂, 6 April 2012; TINOS*: Karya, 1♂, 11 May 2014.

**Distribution in Greece:** Known as *Sarcophaga consanguinea* and *S. portchinskyana* from the provinces of Ioannina, Pieria, Trikala, Attiki, Euboea Isl. and Dodecanese (Rhodes Isl.) [25,26,27,29,30,34,35]. New for the North Aegean Isl. and Cyclades.

**General Distribution:** Algeria, Bulgaria, Croatia, France, Greece, Hungary (?), Israel, Italy, Pakistan, Palestine, Russia, Serbia, Syria, Tunisia, Turkey, and Ukraine [5,30,31,32,33,47].

**Remarks:** Larvae develop as parasitoids of the terrestrial snail *Theba pisana* (O. F. Müller, 1774) [30,53].

***Sarcophaga* (*Heteronychia*) *croca* Pape, 1996**

**Distribution in Greece:** Known as *Sarcophaga croca* and *S. maritima* from the province of Pieria [5,12,24,25,26,29,30].

**General Distribution:** Croatia and Greece [5,30,31,32,33].

***Sarcophaga* (*Heteronychia*) *cucullans* Pandellé, 1896**

**Material Examined:** LIMNOS*: Moudros I, 2♂, 14 May 2012; 3♂, 13 June 2012; Moudros II, 1♂, 14 May 2012; Plaka-Panagia, 10♂, 5 April 2012; 2♂, 13 June 2012.

**Distribution in Greece:** Known from the provinces of Pieria and Trikala [26,27]. New for North Aegean Isl..

**General Distribution:** Armenia, Austria, Azerbaijan, Bosnia and Herzegovina, Bulgaria, Croatia, Czech Republic, France, Greece, Gruzia, Hungary, Israel, Italy, Morocco, Romania, Russia, Serbia, Slovakia, Spain, Switzerland, Turkey, and Ukraine [5,30,31,32,33,47].

**Remarks:** Larvae develop as parasitoids of various terrestrial snails [30].

***Sarcophaga* (*Heteronychia*) *depressifrons* Zetterstedt, 1845**

**Material Examined:** EVROS *: Dadia X, 1♂, 13 August 2012.

**Distribution in Greece:** Known from Ionian Isl. (Corfu Isl.) [29]. New for Thrace.

**General Distribution:** Albania, Austria, Belarus, Belgium, Bosnia and Herzegovina, Bulgaria, China, Croatia, Czech Republic, Denmark, Estonia, Finland, France, Germany, Greece, Hungary, Israel, Italy, Japan, Kazakhstan, Malta, the Netherlands, North Korea, North Macedonia, Norway, Poland, Romania, Russia, Serbia, Slovakia, South Korea, Spain, Sweden, Switzerland, Ukraine, and United Kingdom [5,30,31,32,33].

**Remarks:** It is considered a forest species, but has been reported to inhabit urban environments [2,49].

***Sarcophaga* (*Heteronychia*) *enderleini* Jacentkovský, 1937**

**Distribution in Greece:** Known as *Sarcophaga enderleini* and *S. macedonica* from the provinces of Ioannina, Pieria, Trikala, and Arcadia [5,26,27,29,30].

**General Distribution:** Bulgaria, Greece, and Italy [5,30,31,32].

***Sarcophaga* (*Heteronychia*) *ferox* Villeneuve, 1908** **^▲^ (Figure 2A and Figure 3B)**

**Material Examined:** ATTIKI*: Agia Varvara, 2♂, 13 October 2023; Agricultural University of Athens, 1♂ 26 May 2021; 2♂, 30 May 2021; 1♂, 9 June 2021; 1♂, 8 July 2021; 1♂, 10 August 2021; 1♂, 19 August 2021; 2♂, 27 September 2021; 3♂, 7 October 2021; 1♂, 22 October 2021; Ellinikon International Airport, 2♂, 9 April 2023; CHANIA*: Alikianos, 1♂, 8 May 2023; 1♂, 2 November 2023; 1♂, 26 September 2023; Kefali, 1♂, 28 March 2023; 1♂, 8 May 2023; Lakkoi, 1♂, 2 November 2023; LESVOS*: Castle of Mytilene, 1♂, 1 May 2024; Skala Pamfilon, 1♂, 21 September 2022.

**General Distribution:** Algeria, Canary Isl., Egypt, France, Italy, Malta, Morocco, Spain, Tunisia [5,30,31,32,46]. **New for Greece.**

**Remarks:** At is a heliophilic and coy species, observed to concentrate on rocky hilltops in Sardinia and Sicily where it feeds on the feces of seagulls (Laridae) and birds of prey [57]. It is considered a possible pollinator of *Euphorbia dendroides* L. in Balearic Islands [58].

***Sarcophaga* (*Heteronychia*) *filia* Rondani, 1860**

**Material Examined:** ANAFI*: Helicodrome, 5♂, 12 May 2013; Vagia, 7♂, 13 May 2013; Zoodohos Pigi, 2♂, 12 May 2013; 1♂, 14 May 2013; CHANIA: Alikianos, 1♂, 8 May 2023; 2♂, 26 May 2023; 2♂, 6 July 2023; 1♂, 10 August 2023; 1♂, 1 September 2023; Chania, 1♂, 3 June 2023; CHIOS*: Managros, 1♂, 29 March 2012; EVROS*: Dadia VI, 1♂, 23 September 2012; FOLEGANDROS*: Agios Georgios, 2♂, 13 June 2014; IOS*: Agia Theodoti, 2♂, 16 May 2013; IRAKLEIA*: Irakleia, 1♂, 12 August 2020; Livadi, 1♂, 17 June 2014; LIMNOS*: Plaka-Panagia, 7♂, 5 April 2012; MYKONOS*: Panormos, 1♂, 10 July 2015; SANTORINI*: Agios Fanourios, 1♂, 12 June 2013; Panagia Kalou, 2♂, 9 June 2013; 1♂, 13 June 2013; Pyrgos, 3♂, 8 May 2013; 7♂, 10 June 2013; TINOS*: Karya, 1♂, 27 June 2014.

**Distribution in Greece:** Known from the provinces of Preveza, Thesprotia, Pieria, Larissa, Trikala, Attiki, Crete, and Dodecanese (Rhodes Isl.) [23,25,26,27,29,30,33]. New for Thrace, North Aegean Isl. and Cyclades.

**General Distribution:** Albania, Austria, Belgium, Bulgaria, Croatia, Czech Republic, France, Germany, Greece, Hungary, Israel, Italy, Malta, Moldova, Morocco, the Netherlands, North Macedonia, Palestine, Poland, Romania, Russia, Serbia, Slovakia, Spain, Switzerland, Tunisia, Turkey, Ukraine, and United Kingdom [5,30,31,32,33,47].

**Remarks:** Larvae develop as parasitoids of the terrestrial snails *Cernuella virgata* (Da Costa, 1778), *Helix* spp., and *Theba pisana* (O. F. Müller, 1774) [30,53].

***Sarcophaga* (*Heteronychia*) *haemorrhoa* Meigen, 1826**

**Distribution in Greece:** Known from mainland Greece without specific localities [5,30,31,32,33].

**General Distribution:** Albania, Austria, Azerbaijan, Belarus, Belgium, Bulgaria, Croatia, Czech Republic, Denmark, Estonia, Finland, France, Germany, Greece, Hungary, Ireland, Italy, Latvia, the Netherlands, North Makedonia, Norway, Poland, Romania, Russia, Serbia, Slovakia, Spain, Sweden, Switzerland, Turkey, Ukraine, and United Kingdom [5,30,31,32,33,47].

**Remarks:** Larvae develop as parasitoids of the terrestrial snails *Caucasotachea vindobonensis* (Pfeiffer, 1828), *Cepaea hortensis* (Müller, 1774), and *C. nemoralis* (Linnaeus, 1758) [2,30].

***Sarcophaga* (*Heteronychia*) *haemorrhoides* Böttcher, 1913**

**Distribution in Greece:** Known from the province of Attiki [34,35].

**General Distribution:** Albania, Armenia, Austria, Azerbaijan, Belarus, Belgium, Bulgaria, Croatia, Czech Republic, Estonia, France, Germany, Greece, Gruzia, Hungary, Iran, Iraq, Israel, Italy, Latvia, Malta, Moldova, North Macedonia, Palestine, Poland, Romania, Russia, Sebia, Slovakia, Switzerland, Syria, Turkey, and Ukraine [5,30,31,32,33,47].

**Remarks:** larvae develop as parasitoids of the terrestrial snails *Cantareus apertus* (Born, 1778), *Cepaea nemoralis* (Linnaeus, 1758), and *Eobania vermiculata* (O. F. Müller, 1774) [21,30].

***Sarcophaga* (*Heteronychia*) *helenae* (Trofimov, 1948)**

**Distribution in Greece:** Known from the provinces of Pieria, Larissa, and Trikala [12,29].

**General Distribution:** Armenia, Azerbaijan, Bulgaria, Greece, Gruzia, Israel, Turkey, and Turkmenistan [5,30,31,32,47].

***Sarcophaga* (*Heteronychia*) *hellenica* Whitmore, 2011**

**Distribution in Greece:** Known as *Sarcophaga hellenica* and *S. vervesi* from the provinces of Ioannina, Pieria, and Trikala [25,26,27,29,30].

**General Distribution:** Greece [25,29,30,31,32].

***Sarcophaga* (*Heteronychia*) *infantilis* Böttcher, 1913**

**Distribution in Greece:** Known from the province of Ilia [30].

**General Distribution:** Austria, Bulgaria, Croatia, Czech Republic, France, Greece, Germany, Italy, Kyrgyzstan, Norway, Poland, Serbia, Slovakia, Spain, Sweden, Switzerland, and Turkey [5,30,31,32,33,47].

**Remarks:** Larvae develop as parasitoids of terrestrial snails in the genera *Chondrina* and *Clausilia* [21,30].

***Sarcophaga* (*Heteronychia*) *kataphygionis* (Povolný, 1999)**

**Distribution in Greece:** Known from the province of Pieria [29,30,59].

**General Distribution:** France, Greece, Italy, and Poland [30,31,32,59].

***Sarcophaga* (*Heteronychia*) *kerteszi* Villeneuve, 1912**

**Material Examined:** ANAFI*: Zoodohos Pigi, 1♂, 12 May 2013; CHANIA: Alikianos, 2♂, 8 May 2023; 1♂, 26 May 2023; Kefali, 2♂, 26 May 2023; Lakkoi, 1♂, 1 September 2023; Omalos I, 2♂, 8 May 2023; 1♂, 1 September 2023; 1♂, 26 September 2023; Omalos II, 1♂, 28 March 2023; 1♂, 26 May 2023; 1♂, 10 August 2023; Omalos III, 2♂, 10 August 2023; KARPATHOS*: Avlona, 1♂, 8 June 2012; LESVOS*: Charamida, 1♂, 25 April 2004; 2♂, 11 May 2004; 1♂, 19 May 2004; 2♂, 13 July 2004; Kratigos, 4♂, 18 April 2004; 1♂, 3 June 2004; Loutropoli Thermis, 5♂, 9 September 2022; Mytilene, 2♂, 27 March 2004; 2♂, 24 April 2004; 7♂, 8 May 2004; 3♂, 19 May 2004; 8♂, 8 June 2004; LIMNOS*: Agios Athanasios, 2♂, 14 June 2012; Moudros I, 1♂, 6 April 2012; 8♂, 14 May 2012; 10♂, 13 June 2012; SERIFOS*: Panagia, 1♂, 21 June 2015; TINOS*: Marlas, 1♂, 13 May 2014.

**Distribution in Greece:** Known from the provinces of Attiki (Poros Isl.), Laconia, Ionian Isl. (Corfu), Crete, and Dodecanese (Rhodes Isl.) [5,29,30,31]. New for North Aegean Isl.. Its presence in Cyclades is confirmed (see Remarks).

**General Distribution:** Greece, Israel, Italy, Lebanon, and Turkey [5,30,31,32,47].

**Remarks:** The literature records of this species from Cyclades are without specific localities [31] and probably refer to the Attican island of Poros. After the examination of material from various Cycladic islands (Anafi, Serifos, and Tinos), we are able to confirm the presence of the species in the area.

***Sarcophaga* (*Heteronychia*) *lederbergi* (Lehrer, 1995)**

**Distribution in Greece:** Known as *Sarcophaga ledebergeri* and *S. rohdendorfi* from the province of Pieria [2,26,27,29].

**General Distribution:** Austria, Czech Republic, France, Germany, Greece, Hungary, Italy, Poland, Romania, Russia, Slovakia, Switzerland, and Ukraine [2,5,26,27,30,31,32].

**Remarks:** A locally common species on the slopes of Mt. Olympus [2].

***Sarcophaga* (*Heteronychia*) *minima* Rondani, 1862 (Figure 2J)**

**Material Examined:** ANTIKYTHERA*: Antikythera, 1♂, 4 April 2014; ANYDROS*: Anydros, 1♂, 26 May 2014; ATTIKI: Agia Marina, 1♂, 30 July 2021; Agricultural University of Athens, 3♂, 16.7.202; 1♂ 28 June 2021; 1♂, 30 July 2021; CHANIA: Alikianos, 1♂, 8 May 2023; 2♂, 6 July 2023; Kefali, 2♂, 10 August 2023; 2♂, 1 September 2023; Lakkoi, 1♂, 6 July 2023; DELOS*: Delos, 1♂, 9 July 2015; EVROS*: Dadia IX, 1♂, 22 September 2012; IOS*: Kambos, 1♂, 16 May 2013; IRAKLEIA*: Irakleia, 2♂, 12 August 2020; LESVOS: Antissa I, 1♂, 22 April 2011; Eresos, 1♂, 18 June 2011; Loutra, 1♂, 6 June 2004; Moni Ipsilou I, 1♂, 16 June 2011; Petrified Forest Park “Bali Alonia”, 1♂, 19 June 2011; Sigri I, 2♂, 13 May 2011; Skala Eresou, 1♂, 12 August 2023; LIMNOS*: Plaka-Panagia, 4♂, 5 April 2012; 1♂, 14 May 2012; Moudros I, 1♂, 6 April 2012; RHODES*: Platania, 1♂, 23 May 2012; SANTORINI*: Agios Fanourios, 1♂, 12 June 2013; Panagia Kalou, 1♂, 9 June 2013; SERIFOS*: Megalo Livadi, 3♂, 22 June 2015; TINOS*: Karya, 1♂, 26 June 2014.

**Distribution in Greece:** Known as *Sarcophaga fertoni, S. graeca,* and *S. minima* from the province of Attiki, Mt. Parnassus (no province specified), North Aegean Isl. (Lesvos Isl.), and Crete [5,22,26,29,30,34,35]. New for Thrace, Ionian Isl., Cyclades, and Dodecanese.

**General Distribution:** Algeria, Austria, Bulgaria, Croatia, Czech Republic, Egypt, France, Greece, Hungary, Israel, Italy, Malta, Morocco, Palestine, Portugal, Slovakia, Spain, Tunisia, and Turkey [5,30,31,32,46,47].

**Remarks:** Larvae develop as parasitoids of the terrestrial snails *Cernuella virgata* (Da Costa, 1778), *Cochlicella acuta* (O. F. Müller, 1774), *Theba pisana* (O. F. Müller, 1774), *Trochoidea elegans* (Gmelin, 1791), and *Xerocrassa simulata* (Ehrenberg, 1831) [30,43,53].

***Sarcophaga* (*Heteronychia*) *monspellensia* Böttcher, 1913**

**Distribution in Greece:** Known from the provinces of Attiki (Poros Isl.) and Laconia [5,22,29,31]. Its presence in Cyclades could not be verified (see Remarks).

**General Distribution:** Algeria, France, Greece, Italy, Malta, Spain, Tunisia, and Turkey [5,30,31,32,47].

**Remarks:** The literature records of the species from Cyclades without specific localities [31], are probably associated with the Attican island of Poros. Due to the lack of examined material from the area, we are currently unable to confirm its presence in Cyclades.

***Sarcophaga* (*Heteronychia*) *mutila* Villeneuve, 1912**

**Material Examined:** ANDROS*: Rachi, 1♂, 22 May 2018; IRAKLEIA*: Livadi, 1♂, 17 May 2014; LIMNOS*: Plaka-Panagia, 2♂, 5 April 2012; 5♂, 14 May 2012; 1♂, 13 June 2012; SERIFOS*: Sklavogianni, 1♂, 1 June 2015; TINOS*: Laouti, 1♂, 26 June 2014.

**Distribution in Greece:** the species is found in the provinces of Pieria, Trikala, and Attiki (Poros Island) [26,27,29,30], with new populations from Cyclades.

**General Distribution:** Armenia, Austria, Bulgaria, Croatia, Cyprus, Czech Republic, Greece, Gruzia, Hungary, Italy, Romania, Russia, Serbia, Slovakia, Turkey, and Ukraine [5,30,31,32,33,47].

**Distribution in Greece:** Known from the provinces of Pieria, Trikala, and Attiki (Poros Isl.) [26,27,29,30]. New for Cyclades.

**General Distribution:** Armenia, Austria, Bulgaria, Croatia, Cyprus, Czech Republic, Greece, Gruzia, Hungary, Italy, Romania, Russia, Serbia, Slovakia, Turkey, and Ukraine [5,30,31,32,33,47].

**Remarks:** Larvae are considered parasitoids of helicoid snails [2,30].

***Sarcophaga* (*Heteronychia*) *pandellei* (Rohdendorf, 1937) (Figure 3C)**

**Material Examined:** LIMNOS*: Atsiki, 1♂, 14 June 2012.

**Distribution in Greece:** Known from Ionian Isl. (Corfu Isl.) [33]. New for North Aegean Isl..

**General Distribution:** Algeria, Andorra, Croatia, France, Greece, Italy, Morocco, Poland, Portugal, Spain, and Tunisia [5,30,31,32,33].

***Sarcophaga* (*Heteronychia*) *pauciseta* Pandellé, 1896**

**Distribution in Greece:** Known from mainland Greece without specific localities [2].

**General Distribution:** Austria, Bosnia and Herzegovina, Bulgaria, Croatia, Czech Republic, Estonia, Germany, Greece, Hungary (?), Poland, Russia, Slovakia, Switzerland, and Ukraine [2,5,30,31,32,33].

***Sarcophaga* (*Heteronychia*) *penicillata* Villeneuve, 1907**

**Distribution in Greece:** Known from the province of Pieria [25,26,27].

**General Distribution:** Algeria, Bulgaria, Croatia, France, Greece, Israel, Italy, Malta, Morocco, Spain, and Tunisia [5,30,31,32].

**Remarks:** Records from the province of Attiki [26] constitute misidentifications of *Sarcophaga thirionae* [28]. The species is a recorded parasite of the terrestrial snails *Cochlicella acuta* (O. F. Müller, 1774) and *C. barbara* (Linnaeus, 1758) and exhibits a particular laying method, with the female depositing a single egg in each snail and guarding it for 5–65 min before departing [30,60].

***Sarcophaga* (*Heteronychia*) *porrecta* Böttcher, 1913**

**Distribution in Greece:** Known from the provinces of Ioannina and Pieria [2,25,26,27,29].

**General Distribution:** Austria, Bulgaria, Croatia, Czech Republic, Greece, Italy, Romania, Serbia, Slovakia, and Turkey [5,30,31,32,33,47].

**Remarks:** it is a strictly montane species [2].

***Sarcophaga* (*Heteronychia*) *pseudobenaci* (Baranov, 1942)**

**Distribution in Greece:** Known from the province of Thessaloniki [29].

**General Distribution:** Bulgaria, Croatia, Greece, Romania, and Serbia [30,31,32,33].

***Sarcophaga* (*Heteronychia*) *pumila* Meigen, 1826**

**Distribution in Greece:** Known from the province of Pieria [26,27].

**General Distribution:** Austria, Belgium, Bulgaria, Croatia, Czech Republic, Denmark, Estonia, Finland, France, Germany, Greece, Hungary, Israel, Italy, Latvia, Lithuania, the Netherlands, Norway, Poland, Romania, Russia, Slovakia, Spain, Sweden, Switzerland, Ukraine, and United Kingdom [5,30,31,32,33].

**Remarks:** Larvae develop as parasitoids of the terrestrial snails *Cernuella virgata* (Da Costa, 1778) and *Theba pisana* (O. F. Müller, 1774) [30].

***Sarcophaga* (*Heteronychia*) *rondaniana* (Rohdendorf, 1937)**

**Material Examined:** CHANIA*: Omalos I, 1♂, 26 September 2023; LESVOS*: Pirgi Thermis; 1♂, 15 October 2020; LIMNOS*: Moudros II, 2♂, 13 June 2012.

**Distribution in Greece:** Known from the province of Pieria [26,27]. New for North Aegean Isl. and Crete.

**General Distribution:** Austria, Bulgaria, Croatia, Czech Republic, France, Germany, Greece, Hungary, Italy, the Netherlands, North Macedonia, Romania, Serbia, Slovakia, Spain, Switzerland, Turkey, and Ukraine [5,30,31,32,33,47].

***Sarcophaga* (*Heteronychia*) *schineri* Bezzi, 1891**

**Distribution in Greece:** Known from the province of Pieria [26,27].

**General Distribution:** Albania, Andorra, Armenia, Austria, Azerbaijan, Bulgaria, Croatia, Czech Republic, France, Germany, Greece, Gruzia, Hungary, Italy, North Macedonia, Poland, Romania, Russia, Serbia, Slovakia, Switzerland, Turkey, and Ukraine [2,5,30,31,32,33,47].

***Sarcophaga* (*Heteronychia*) *setinervis* Rondani, 1860**

**Material Examined:** CHANIA*: Omalos II, 1♂, 10 August 2023; LESVOS*: Castle of Mytilene, 3♂, 27 March 2024; MYKONOS*: Panormos, 1♂, 10 July 2015; TINOS*: Karya, 1♂, 26 June 2014.

**Distribution in Greece:** Known from the provinces of Pieria and Trikala [25,26,27]. New for North Aegean Isl., Cyclades, and Crete.

**General Distribution:** Armenia, Austria, Azerbaijan, Bulgaria, Cyprus, Egypt, France, Greece, Gruzia, Hungary, Iran, Israel, Italy, Jordan, Kazakhstan, Kyrgyzstan, North Macedonia, Palestine, Romania, Serbia, Sudan (?), Tajikistan, Turkey, Turkmenistan, and Uzbekistan [5,30,31,32,46,47].

***Sarcophaga* (*Heteronychia*) *siciliensis* Böttcher, 1913**

**Distribution in Greece:** Known from the province of Pieria [25,26,27].

**General Distribution:** Bulgaria, Canary Isl., Cyprus, Egypt, Greece, Hungary, Israel, Italy, Palestine, Romania, Spain, Syria, Turkey, and Ukraine [5,30,31,32,46,47].

***Sarcophaga* (*Heteronychia*) *thirionae* (Lehrer, 1976) (Figure 2C)**

**Material Examined:** ATTIKI: Agia Marina, 2♂, 3 April 2022 and 26 March 2023; CHANIA: Alikianos, 1♂, 8 May 2023; 1♂, 6 July 2023; IRAKLEIO: Irakleio, 1♂, 10 October 2015.

**Distribution in Greece:** Known from the province of Attiki and Crete [25,28,30].

**General Distribution:** Algeria, France, Italy, Bulgaria, Greece, and Turkey [5,30,31,32,47].

***Sarcophaga* (*Heteronychia*) *vicina* Macquart, 1835**

**Distribution in Greece:** Known from the province of Trikala [26,27].

**General Distribution:** Austria, Bosnia and Herzegovina, Bulgaria, Croatia, Czech Republic, Finland, France, Germany, Greece, Gruzia, Hungary, Ireland, Italy, Norway, Poland, Russia, Serbia, Slovakia, Spain, Sweden, Switzerland, Ukraine, and United Kingdom [5,30,31,32,33].

**Remarks:** Larvae develop as parasitoids of terrestrial snails [2,30,53].

***Sarcophaga* (*Krameromyia*) *anaces* Walker, 1849** **^▲^ (Figure 3D)**

**Material Examined:** CHANIA*: Alikianos, 2♂, 8 May 2023; 1♂, 26 May 2023.

**General Distribution:** Algeria, Armenia, Austria, Belgium, Bulgaria, Croatia, Czech Republic, France, Germany, Hungary, Italy, the Netherlands, Poland, Portugal, Slovakia, Spain, Switzerland, Turkey, Ukraine, and United Kingdom [5,31,32,33,47]. **New for Greece.**

***Sarcophaga* (*Latistyla*) *czernyi* Böttcher, 1912 (Figure 3E)**

**Material Examined:** LESVOS*: Mytilene, 1♂, 8 May 2004.

**Distribution in Greece:** Known from mainland Greece without specific localities [5,31,32,33,44]. New for North Aegean Isl..

**General Distribution:** Croatia, Greece, Israel, Lebanon, and Turkey [5,31,32,33,44,47].

***Sarcophaga* (*Liopygia*) *argyrostoma* (Robineau-Desvoidy, 1830) (Figure 2E)**

**Material Examined:** ATTIKI: Agricultural University of Athens, 1♂, 19 July 2021; CHANIA*: Chania, 1♂, 7 June 2023; EVROS*: Dadia V, 1♂, 22 September 2012; IRAKLEIA*: Irakleia, 4♂, 12 August 2020; LESVOS*: Kechrada, 1♂, 15 July 2023; Palios, 1♂, 31 July 2022; 1♂, 30 September 2023; Pirgi Thermis, 1♂, 2 July 2022; 1♂. 16 September 2022; SYROS*: 1♂ 11 September 2023.

**Distribution in Greece:** Known from the provinces of Ioannina, Thesprotia, Pieria, Trikala, and Attiki [23,26,27]. New for Thrace, North Aegean Isl., Cyclades, and Crete.

**General Distribution:** Afghanistan, Albania, Argentina, Armenia, Austria, Azerbaijan, Azores, Belgium, Bermuda, Brazil, Bulgaria, Canada, the Canary Isl., China, Croatia, Cuba, Cyprus, Czech Republic, Denmark, Egypt, France, French Polynesia, Germany, Greece, Gruzia, Hawaiian Isl., Hungary, India, Iran, Iraq, Israel, Italy, Kazakhstan, Kyrgyzstan, Latvia, Madeira, Malta, Marshall Isl., Moldova, Mongolia, Morocco, Netherlands, North Macedonia, Pakistan, Poland, Portugal, Romania, Russia, Saint Helena, Saudi Arabia, Serbia, Slovakia, South Africa, Spain, Switzerland, Syria, Tajikistan, Tunisia, Turkey, Turkmenistan, Ukraine, United Kingdom, Uruguay, USA, Uzbekistan, and Wake Isl. [5,31,32,33,46,47].

**Remarks:** It is a culturophilic and synanthropic species [2,49]. Larvae develop in a plethora of decaying organic substrates, including feces and carcasses, and are considered predators and parasitoids of terrestrial snails, acridoid grasshoppers (both adults and oothecae), Coleoptera, Lepidoptera (both larvae and pupae), and larvae of other saprophagous Diptera [2,21,43,51]. The species is a known agent of myiasis in sheep and humans [2,43,61].

***Sarcophaga* (*Liopygia*) *crassipalpis* Macquart, 1839 (Figure 2B)**

**Material Examined:** ACHAIA*: Ano Platanos Akratas, 2♂, 12 July 2018; ATTIKI: Agricultural University of Athens, 1♂, 22 June 2021; 1♂, 30 July 2021; 1♂, 21 September 2021; CHANIA*: Hora Sfakion, 1♂, 4 June 2023; LESVOS*: Anemotia, 1♂, 25 September 2023; Castle of Mytilene, 3♂, 21 April 2023; 1♂, 27 March 2024; Pirgi Thermis, 1♂, 21 September 2023; 1♂, 23 October 2023; 1♂, 28 October 2023; LIMNOS*: Atsiki, 1♂, 15 May 2012.

**Distribution in Greece:** Known from the provinces of Preveza, Thesprotia, Pieria, Trikala, and Attiki [23,26,27]. New for Peloponnese, North Aegean Isl. and Crete.

**General Distribution:** Afghanistan, Albania, Algeria, Argentina, Armenia, Australia, Austria, Azerbaijan, Azores, Bulgaria, Canada, Canary Isl., Chile, China, Croatia, Cyprus, Czech Republic, Egypt, France, French Polynesia, Germany, Greece, Gruzia, Hungary, Iran, Iraq, Israel, Italy, Japan, Kazakhstan, Kyrgyzstan, Lebanon, Libya, Madeira, Malta, Marshall Isl., Moldova, Mongolia, Morocco, New Zealand, North Korea, Papua New Guinea, Portugal, Romania, Russia, Saudi Arabia, Serbia, Slovakia, South Africa, South Korea, Spain, Syria, Tajikistan, Thailand, Tunisia, Turkey, Turkmenistan, Ukraine, Uruguay, USA, and Uzbekistan [5,31,32,33,46,47].

**Remarks:** It is a culturophilic and synanthropic species [2]. Larvae develop in decaying meat and carcasses of both vertebrate and invertebrate origin, as well as in the oothecae of acridoid grasshoppers [2,21,43,51]. It is a species of forensic and medical importance and is known to cause cutaneous myiasis in sheep and the Agamid lizard *Saara hardwicki* (Gray, 1827) and aural, intestinal, and wound myiasis in humans [2,21,32,43,61].

***Sarcophaga* (*Liosarcophaga*) *dux* Thomson, 1869**

**Material Examined:** ANAFI*: Vagia, 2♂, 13 May 2013.

**Distribution in Greece:** Known from the provinces of Thesprotia and Pieria [23,26,27]. New for Cyclades.

**General Distribution:** Albania, Andaman Isl., Australia, Azerbaijan, Azores, Bangladesh, Bhutan, Bonin Isl., Bulgaria, Canary Isl., Cape Verde Isl., China, Christmas Isl., Croatia, Cyprus, Egypt, Fiji, France, Greece, Gruzia, Guam, Hawaiian Isl., India, Indonesia, Israel, Italy, Japan, Kazakhstan, Kiribati, Kosovo, Libya, Lord Howe Isl., Macedonia, Malaysia, Malta, Marshall Isl., Micronesia, Montenegro, Morocco, Nepal, New Caledonia, Niue (?), Qatar, Pakistan, Palau, Philippines, Romania, Saudi Arabia, Serbia, Solomon Isl., South Korea, Singapore, Spain, Sri Lanka, Sudan, Switzerland, Taiwan, Thailand, Turkmenistan, Vanuatu, Vietnam, Ukraine, United Arab Emirates, Wake Isl., Western Samoa [5,31,32,33,46,47,48].

**Remarks:** Larvae develop in feces, a wide variety of vertebrate and invertebrate carcasses, and sometimes garbage inside buildings [13,21,43,49,51]. The species is of significant medical (agent of myiasis) and forensic importance [62].

***Sarcophaga* (*Liosarcophaga*) *emdeni* (Rohdendorf, 1969)**

**Material Examined:** ANAFI*: Vagia, 3♂, 13 May 2013; EVROS*: Dadia VIII, 1♂, 22 September 2012; IRAKLEIA*: Irakleia, 2♂, 12 August 2020; Livadi, 1♂, 19 March 2014; 1♂, 17 May 2014; Pigadi Beach, 1♂, 19 May 2014; SANTORINI*: Panagia Kalou, 2♂, 5 April 2013.

**Distribution in Greece:** Known from the provinces of Ioannina, Pieria, and Trikala [26,27]. New for Thrace and Cyclades.

**General Distribution:** Austria, Azerbaijan, Bulgaria, China, Croatia, Czech Republic, Denmark, Estonia, Finland, Germany, Greece, Gruzia, Hungary, Iran, Italy, Kazakhstan, Kyrgyzstan, Norway, Poland, Romania, Russia, Slovakia, Sweden, Switzerland, Turkey, and Ukraine [5,31,32,33,47].

**Remarks:** Larvae are considered necrophagous and facultative parasitoids of the larvae of Lepidoptera [2].

***Sarcophaga* (*Liosarcophaga*) *harpax* Pandellé, 1896**

**Distribution in Greece:** Known from mainland Greece without specific localities [32].

**General Distribution:** Austria, Azerbaijan, Bangladesh, Belarus, Bulgaria, China, Croatia, Czech Republic, France, Germany, Greece, Gruzia, Hungary, India, Italy, Japan, Kazakhstan, Kyrgyzstan, Latvia, Moldova, Mongolia, the Netherlands, North Korea, Poland, Romania, Russia, Serbia, Slovakia, South Korea, Sri Lanka, Tajikistan, and Ukraine [5,31,32,33].

**Remarks:** Larvae develop as parasitoids of arthropods (mainly insects), including pupae of Lepidoptera, and possibly as predators of larvae of saprophagous Diptera in carcasses [2,43]. The species is reported as an occasional cause of myiasis [2,43].

***Sarcophaga* (*Liosarcophaga*) *jacobsoni* (Rohdendorf, 1937)**

**Material Examined:** ATTIKI*: Agricultural University of Athens, 1♂, 24 May 2021; 1♂, 8 July 2021; 2♂, 16 July 2021; 1♂, 23 July 2021; 1♂, 8 September 2021; 8♂, 18 October 2021; 1♂, 5 November 2021; LESVOS*: Castle of Mytilene, 1♂, 9 April 2006; Petra, 1♂, 23 September 2023; Skala Eresou, 1♂, 12 August 2023; Skala Pamfilon, 1♂, 18 April 2022.

**Distribution in Greece:** Known from the provinces of Preveza, Thesprotia, Pieria, and Trikala [23,26,27]. New for Sterea Ellada and North Aegean Isl.

**General Distribution:** Albania, Algeria, Armenia, Azerbaijan, Azores, Bulgaria, Canary Isl., China, Croatia, Cyprus, Czech Republic, Denmark, Egypt, France, Germany, Greece, Gruzia, Hungary, Iran, Ireland, Israel, Italy, Kazakhstan, Malta, Moldova, Mongolia, Morocco, North Korea, Romania, Russia, Slovakia, Spain, Tajikistan, Tunisia, Turkey, Turkmenistan, Ukraine, United Kingdom, and Uzbekistan [5,31,32,33,46,47].

**Remarks:** The species shows culturophilic and synanthropic tendencies [2]. Larvae develop on carcasses (both from vertebrates and invertebrates) and feces and are predators of the larvae of other saprophagous Diptera [2,21,43]. It is also a known passive vector of protozoan cysts [2].

***Sarcophaga* (*Liosarcophaga*) *portschinskyi* (Rohdendorf, 1937)**

**Material Examined:** CHANIA*: Kefali, 1♂, 26 May 2023; Lakkoi, 1♂, 26 May 2023; Omalos III, 1♂, 6 July 2023; EVROS*: Dadia IV, 1♂, 22 September 2012; Dadia V, 1♂, 22 September 2012; Dadia VII, 1♂, 23 September 2012; KARPATHOS*: Avlona, 1♂, 8 June 2012; LESVOS*: Pelopi, 1♂, 15 July 2023; Sanatorio Agiasou, 1♂, 8 July 2023.

**Distribution in Greece:** Known from the provinces of Ioannina, Preveza, Thesprotia, Pieria, and Trikala [23,26,27]. New for Thrace, North Aegean Isl., Crete, and Dodecanese.

**General Distribution:** Albania, Algeria, Andorra, Armenia, Austria, Azerbaijan, Belarus, Belgium, Bulgaria, China, Croatia, Czech Republic, Denmark, Estonia, Finland, France, Germany, Greece, Gruzia, Hungary, Ireland, Italy, Kazakhstan, Kyrgyzstan, Latvia, Malta, Moldova, Mongolia, the Netherlands, Norway, Pakistan, Poland, Portugal, Romania, Russia, Slovakia, Sweden, Switzerland, Tajikistan, Turkey, Turkmenistan, Ukraine, and Uzbekistan [5,31,32,33,47].

**Remarks:** It is a xerophilic species with culturophilic tendencies [2]. Larvae are reported as coprophagous and as predators of muscoid larvae in feces and carcasses [2,55]. Also, it develops in a variety of organic substrates, including living terrestrial snails, Lepidoptera (larvae and pupae), Coleoptera, and Orthoptera, and on vertebrate carcasses [2,21,43].

***Sarcophaga* (*Liosarcophaga*) *tibialis* Macquart, 1850 (Figure 2K)**

**Material Examined:** ATTIKI*: Agia Varvara, 1♂, 13 October 2023; Agricultural University of Athens, 1♂, 26 May 2021; 1♂, 9 June 2021; 2♂, 14 June 2021; 2♂, 8 July 2021; 1♂, 13 July 2021; 3♂, 16 July 2021; 1♂, 19 July 2021; 1♂, 10 August 2021; 1♂, 7 October 2021; 8♂, 18 October 2021; Ellinikon International Airport, 1♂, 3 January 2023; CHANIA*: Alikianos, 1♂, 26 September 2023; 3♂, 2 November 2023; Omalos II, 1♂, 10 August 2023; KORINTHIA: Lechaio, 1♂, 3 July 2022; 1♂, 28 July 2022; 1♂, 11 June 2023; LESVOS*: Pirgi Thermis, 1♂, 24 May 2023; LIMNOS*: Moudros I, 1♂, 14 May 2012; NAXOS*: Potamia, 2♂, 29 July 2019.

**Distribution in Greece:** Known from the provinces of Preveza, Thesprotia, Pieria, Trikala, and Messinia [23,26,27,36]. New for Sterea Ellada, North Aegean Isl., Cyclades, and Crete.

**General Distribution:** Algeria, Angola, Botswana, Bulgaria, Burkina Faso, Cameroon, Canary Isl., Chagos Archipelago, Croatia, Cyprus, Czech Republic, Egypt, Ethiopia, France, French Polynesia, Greece, Indonesia, Italy, Ivory Coast, Lesotho, Liberia, Madagascar, Madeira, Malta, Morocco, Mozambique, Namibia, New Caledonia, Nigeria, Oman, Saudi Arabia, Réunion, Seychelles, Sierra Leone, Somalia, South Africa, Spain, Sudan, Tanzania, Togo, Tunisia, Turkey, Yemen, Zaire, Zambia, and Zimbabwe [5,31,32,33,46,47].

**Remarks:** It is a thermophilic and heliophilic species with strong culturophilic and synanthropic tendencies [2]. Larvae develop in a variety of organic substrates, including living Orthoptera, turtle eggs, a myxomatosed rabbit, and carcasses [2,21,43,51]. The species causes myiasis, and a larva has been collected from a human wound [2,43,61].

***Sarcophaga* (*Liosarcophaga*) *tuberosa* Pandellé, 1896**

**Distribution in Greece:** Known from mainland Greece without specific localities [5,31,32,33].

**General Distribution:** Austria, Azerbaijan, Belgium, Bulgaria, China, Croatia, Czech Republic, Egypt (?), France, Germany, Greece, Gruzia, Hungary, Italy, Japan, Kazakhstan, Kyrgyzstan, Mongolia, Morocco, Netherlands, Pakistan, Poland, Romania, Russia, Serbia, Slovakia, South Korea, Spain, Switzerland, Taiwan, Tajikistan, Turkmenistan, Turkey, Ukraine, and Uzbekistan [5,31,32,33,46,47,48].

**Remarks:** Larvae are considered predators or parasitoids of the pupae of Lepidoptera, but are able to develop in other insects and snails, too [2,21,43]. Reported to cause cutaneous myiasis in humans [2,43,61].

***Sarcophaga* (*Mehria*) *sexpunctata* (Fabricius, 1805)**

**Distribution in Greece:** Known from mainland Greece without specific localities [5,31,32,33].

**General Distribution:** Armenia, Austria, Belgium, Bulgaria, Canary Isl., China, Croatia, Czech Republic, Denmark, Finland, France, Germany, Greece, Hungary, Ireland, Italy, Japan, Kazakhstan, Mongolia, the Netherlands, Norway, Poland, Romania, Russia, Slovakia, Spain, Sweden, Switzerland, Turkey, Ukraine, and United Kingdom [5,31,32,33,47].

**Remarks:** It is a predator of egg sacs of spiders in the families Araneidae [*Araneus* spp. and *Larinioides cornutus* (Clerck, 1757)] and Clubionidae (*Clubiona* spp.) [2,43]. Breeding records from acridoid grasshoppers require confirmation [43].

***Sarcophaga* (*Myorhina*) *nigriventris* Meigen, 1826**

**Material Examined:** ANAFI*: Helicodrome, 1♂, 13 April 2013; 3♂, 12 May 2013; Vagia, 13♂, 13 May 2013; Zoodohos Pigi, 1♂, 12 May 2013; CHANIA*: Alikianos, 2♂, 8 May 2023; 4♂, 26 May 2023; 1♂, 26 September 2023; Kefali, 1♂, 8 May 2023; 1♂, 10 August 2023; Lakkoi, 1♂, 26 May 2023; Omalos I, 3♂, 26 May 2023; Omalos II, 1♂, 6 July 2023; Omalos III, 1♂, 6 July 2023; FOLEGANDROS*: Agios Georgios, 1♂, 12 June 2014; LESVOS*: Castle of Mytilene, 4♂, 27 March 2024; SANTORINI*: Panagia Kalou, 1♂, 9 June 2013; Pyrgos, 1♂, 8 May 2013.

**Distribution in Greece:** Known from the provinces of Ioannina, Preveza, Thesprotia, Pieria, and Trikala [23,26,27]. New for North Aegean Isl., Cyclades, and Crete.

**General Distribution:** Albania, Algeria, Andorra, Armenia, Austria, Azerbaijan, Belgium, Bulgaria, Croatia, Cyprus, Czech Republic, Denmark, Ireland, France, Germany, Greece, Gruzia, Hungary, Ireland, Italy, Malta, Morocco, the Netherlands, Poland, Portugal, Romania, Russia, Serbia, Slovakia, Spain, Switzerland, Tunisia, Turkey, Ukraine, and United Kingdom [5,31,32,33,47].

**Remarks:** A very adaptable species recorded from dry sunlit localities, as well as marshy, sandy, and pond habitats [2,50]. Larvae are considered facultative parasitoids of a number of terrestrial snail species [2,43,53]. It has been bred from living and dead Coleoptera (adults and larvae), bees, and acridoid grasshoppers and from vertebrate carcasses [2,21,43].

***Sarcophaga* (*Myorhina*) *socrus* Rondani, 1860**

**Material Examined:** CHANIA*: Kefali, 14♂, 28 March 2023; 2♂, 26 May 2023; 2♂, 8 May 2023; 1♂, 2 November 2023; Omalos I, 1♂, 26 May 2023; Omalos II, 5♂, 28 March 2023; 2♂, 26 May 2023; CHIOS*: Emporios, 2♂, 18 June 2013; EVROS*: Dadia VII, 1♂, 23 September 2012; KORINTHIA*: Lechaio, 2♂, 22 April 2024; LESVOS*: Agia Marina, 1♂, 15 October 2017; Castle of Mytilene, 1♂, 25 April 2023; 23♂, 27 March 2024; Mistegna, 1♂, 21 May 2020; Plaka Park, 1♂, 27 May 2011; Sigri I, 14♂, 13 May 2011; Sigri II, 1♂, 24 May 2011; LIMNOS*: Agios Athanasios, 1♂, 14 June 2012; Moudros I, 2♂, 14 May 2012; Moudros II, 1♂, 14 May 2012.

**Distribution in Greece:** Known from the provinces of Ioannina, Pieria, and Trikala [26,27]. New for Thrace, Peloponnese, North Aegean Isl. and Crete.

**General Distribution:** Albania, Andorra, Austria, Bulgaria, Croatia, Czech Republic, Estonia, Finland, France, Germany, Greece, Hungary, Italy, Poland, Russia, Slovakia, Switzerland, Turkey, and Ukraine [5,31,32,33,47].

**Remarks:** A heliophilic species, also reported from sea shores [2].

***Sarcophaga* (*Myorhina*) *soror* Rondani, 1861**

**Distribution in Greece:** Known from the provinces of Ioannina, Pieria, and Trikala [26,27].

**General Distribution:** Austria, Azerbaijan, Bulgaria, Canary Isl., Croatia, Czech Republic, Denmark, Estonia, Finland, France, Germany, Greece, Gruzia, Hungary, Ireland, Italy, Morocco, Norway, Poland, Romania, Russia, Slovakia, Spain, Sweden, Switzerland, Turkey, Ukraine, and United Kingdom [5,26,27,31,32,33,47].

**Remarks:** Reported as a facultative parasitoid of the snail *Cornu aspersum* (O. F. Müller, 1774) [53].

***Sarcophaga* (*Pandelleana*) *protuberans* Pandellé, 1896**

**Material Examined:** IRAKLEIA*: Irakleia, 1♂, 12 August 2020.

**Distribution in Greece:** Known from the provinces of Ioannina, Pieria, and Trikala [26,27]. New for Cyclades.

**General Distribution:** Armenia, Austria, Azerbaijan, Bulgaria, China, Croatia, Cyprus, Czech Republic, France, Germany, Greece, Gruzia, Hungary, Italy, Kazakhstan, Moldova, Morocco, the Netherlands, Poland, Romania, Russia, Serbia, Slovakia, Spain, Switzerland, Turkey, and Ukraine [5,26,27,31,32,33,47].

**Remarks:** A predator of lizard eggs (Lacertidae) [43].

***Sarcophaga* (*Parasarcophaga*) *albiceps* Meigen, 1826**

**Distribution in Greece:** Known from the provinces of Ioannina, Thesprotia, Pieria, and Trikala [23,26,27].

**General Distribution:** Albania, Andaman Isl., Armenia, Australia, Austria, Azerbaijan, Bangladesh, Belarus, Belgium, Bhutan, Bulgaria, China, Croatia, Czech Republic, Finland, France, Germany, Greece, Gruzia, Hawaiian Isl., Hungary, India, Indonesia, Israel, Italy, Japan, Kazakhstan, Kenya, Laccadive Isl., Latvia, Malaysia, Malta, Moldova, Nepal, the Netherlands, North Korea, Norway, Pakistan, Papua New Guinea, Philippines, Poland, Portugal, Romania, Russia, Serbia, Singapore, Slovakia, Solomon Isl., South Korea, Spain, Sri Lanka, Sweden, Switzerland, Taiwan, Thailand, Turkey, Vietnam, Ukraine, and United Kingdom [5,31,32,33,47].

**Remarks:** A common, widespread culturophilic and synanthropic species, frequently collected from areas bordering ponds and lakes [2,33]. It breeds almost exclusively in feces when given the choice (coprobiodotic) but has also been recorded to larviposit on other types of decaying matter and carrion, including mutton and fish [2,13,43,63,64]. Larvae are considered facultative predators of a variety of Lepidoptera (both larvae and pupae), Coleoptera and the sawfly *Acantholyda posticalis* (Matsumura, 1912), and occasional predators of the larvae of other saprophagic Diptera [2,21,43]. Known to cause myiasis in cattle and humans [2,43,61].

***Sarcophaga* (*Phytosarcophaga*) *destructor* Malloch, 1929**

**Distribution in Greece:** Known from mainland Greece without specific localities [5,31,32,33].

**General Distribution:** Algeria, Burkina Faso, Canary Isl., Croatia, Cyprus, Djibouti, Egypt, Eritrea, Ethiopia, France, Greece, Iraq, Israel, Italy, Mali, Malta, Saudi Arabia, Somalia, Spain, Sudan, Syria, Tanzania, Turkey, Turkmenistan, Uganda, United Arab Emirates, and Yemen [5,31,32,33,46,47].

**Remarks:** The species is probably able to develop entirely on decomposing vegetable matter, as noted from numerous breeding records associated with the pulp of melons and tomatoes [43]. It has also been reported to visit faces from humans and (other) animals and has been bred from dying or moribund acridoid grasshoppers [50,65,66].

***Sarcophaga* (*Pseudothyrsocnema*) *spinosa* Villeneuve, 1912**

**Distribution in Greece:** Known from the provinces of Preveza and Pieria [26,27].

**General Distribution:** Albania, Azerbaijan, Egypt, France, Greece, Gruzia, Hungary, Italy, Macedonia, Romania, Russia (South European Territory), Serbia, Syria, Turkey, Turkmenistan, and Ukraine [5,26,27,31,32,46,47].

***Sarcophaga* (*Rosellea*) *beckiana* (Lehrer, 1996)** **^▲^ (Figure 2F and Figure 3F)**

**Material Examined:** LESVOS*: Pirgi Thermis, 1♂, 25 April 2022.

**General Distribution:** Israel and Turkey [32,47,67]. New for Europe and Greece.

***Sarcophaga* (*Sarcophaga*) *carnaria* (Linnaeus, 1758)**

**Distribution in Greece:** Known as *S. schultzi* from the province of Pieria [26,27].

**General Distribution:** Armenia, Austria, Azerbaijan, Belarus, Belgium, Bulgaria, Croatia, Czech Republic, Denmark, Estonia, Finland, France, Germany, Greece, Gruzia, Hungary, Ireland, Italy, Kazakhstan, Latvia, Lithuania, Luxembourg, Moldova, the Netherlands, Norway, Poland, Romania, Russia, Slovakia, Sweden, Switzerland, Turkey, Ukraine, and United Kingdom [5,31,32,33,47].

**Remarks:** Considered very rare and at the limit of its ecological range in Greece [26]. The larvae are reported in older literature breeding in a variety of organic substrates, but are now considered obligate predators of earthworms (Lumbricina) [43].

***Sarcophaga* (*Sarcophaga*) *lehmanni* Müller, 1922 (Figure 2H)**

**Material Examined:** ARCADIA*: Tripoli, 1♂, 26 March 2023; ATTIKI*: Agia Marina, 2♂, 11 December 2022; Agia Varvara, 1♂, 6 January 2023; 1♂, 13 April 2023; Agricultural University of Athens, 1♂, 22 June 2021; 2♂, 13 July 2021; 1♂, 16 July 2021; Ellinikon International Airport, 2♂, 12 March 2023; Ippokrateios Politeia, 1♂, 4 May 2023; CHANIA*: Alikianos, 1♂, 28 March 2023; 3♂, 8 May 2023; 2♂, 26 May 2023; 6♂, 2 November 2023; Kefali, 23♂, 28 March 2023; 2♂, 8 May 2023; 3♂, 26 May 2023; Lakkoi, 1♂, 26 September 2023; Omalos II, 1♂, 28 March 2023; 2♂, 8 May 2023; 2♂, 26 May 2023; 1♂, 26 September 2023; Omalos III, 1♂, 8 May 2023; 1♂, 26 May 2023; 2♂, 1 September 2023; 1♂, 26 September 2023; 1♂, 2 November 2023; EVROS*: Dadia III, 1♂, 23 August 2012; KORINTHIA*: Doxa Lake, 1♂, 10 April 2022; Kato Trikala, 1♂, 17 April 2019; LESVOS*: Antissa I, 2♂, 22 April 2011; Archaia Antissa, 1♂, 30 April 2023; Karava, 1♂, 9 June 2012; Kremasti Bridge, 1♂, 12 October 2022; Latomeio Eresou, 2♂, 29 April 2011; Latomeio Pigis, 1♂, 22 May 2023; Moni Ipsilou I, 1♂, 5 May 2011; Palios, 1♂, 30 September 2023, Pamfila, 1♂, 25 September 2023; Antissa II, 1♂, 19 May 2011; Pelopi, 1♂, 15 July 2023; Pirgi Thermis, 1♂, 15 October 2020; 1♂, 28 April 2023; 3♂, 29 April 2023; 1♂, 13 July 2023; Sanatorio, 1♂, 8 July 2023; 2♂, 13 July 2024; Sigri I, 1♂, 13 May 2011; Skala Pamfilon, 2♂, 25 May 2023; Vatousa, 1♂, 16 May 2011; LIMNOS*: Atsiki, 1♂, 15 May 2012; Moudros I, 2♂, 14 May 2012; Moudros II, 3♂, 6 April 2012; 1♂, 14 May 2012; 4♂, 13 June 2012; Plaka-Panagia, 1♂, 5 April 2012.

**Distribution in Greece:** Known as *Sarcophaga lasiostyla* and *S. lehmanni* from the provinces of Ioannina, Preveza, Thesprotia, Pieria, and Trikala [23,26,27]. New for Thrace, Sterea Ellada, Peloponnese, North Aegean Isl. and Crete.

**General Distribution:** Afghanistan, Albania, Algeria, Andorra, Armenia, Austria, Azerbaijan, Belarus, Belgium, Bulgaria, Croatia, Cyprus, Czech Republic, Denmark, Egypt, Estonia, France, Germany, Greece, Gruzia, Hungary, Iran, Iraq, Israel, Italy, Jordan, Kazakhstan, Kyrgyzstan, Latvia, Lithuania, Malta, Moldova, Morocco, the Netherlands, Norway, Poland, Portugal, Romania, Russia, Saudi Arabia, Slovakia, Spain, Sweden, Switzerland, Tajikistan, Tunisia, Turkey, Turkmenistan, Ukraine, and Uzbekistan [5,31,32,33,46,47].

**Remarks:** Adults prefer warm, sunlit habitats in or near forests and have been reported around ponds [2,50]. They are attracted to meat and feces (Rognes 1986). Larvae are considered predators of earthworms (Lumbricina) [2,43].

***Sarcophaga* (*Sarcophaga*) *pagensis* Baranov, 1939**

**Distribution in Greece:** Known from the provinces of Pieria and Trikala [26,27].

**General Distribution:** Croatia, France, and Greece [5,31,32,33].

***Sarcophaga* (*Sarcophaga*) *variegata* (Scopoli, 1763)**

**Distribution in Greece:** Known from the provinces of Pieria and Trikala [26,27].

**General Distribution:** Albania, Algeria, Andorra, Austria, Belarus, Belgium, Bulgaria, China, Croatia, Czech Republic, Denmark, Estonia, Finland, France, Germany, Greece, Gruzia, Hungary, Italy, Kazakhstan, Latvia, Lithuania, Luxembourg, Moldova, Mongolia, Montenegro, the Netherlands, Norway, Poland, Portugal, Romania, Russia, Serbia, Slovakia, Spain, Sweden, Switzerland, Tajikistan, Turkey, Ukraine, and United Kingdom [5,31,32,33,47].

**Remarks:** The species is considered very rare and at the limit of its ecological range in Greece [26]. Larvae are considered obligate predators of earthworms (Lumbricina) but are also recorded to develop on Lepidoptera pupae and snails [2,43]. Reports of myiasis in vertebrates need verification [2,61].

***Sarcophaga* (*Stackelbergeola*) *mehadiensis* Böttcher, 1912**

**Distribution in Greece:** Known from mainland Greece without specific localities [31,32,33].

**General Distribution:** Armenia, Azerbaijan, Croatia, Czech Republic, France, Greece, Romania, and Turkey [5,31,32,33,47].

***Sarcophaga* (*Thyrsocnema*) *incisilobata* Pandellé, 1896 (Figure 2L)**

**Material Examined:** CHANIA*: Chania, 1♂, 3 June 2023; Kefali, 2♂, 28 March 2023; 4♂, 8 May 2023; Lakkoi, 2♂, 28 March 2023; 1♂, 8 May 2023; 2♂, 26 May 2023; 1♂, 26 September 2023; Omalos I, 1♂, 8 May 2023; 2♂, 26 May 2023; Omalos II, 1♂, 8 May 2023; 1♂, 26 May 2023; 2♂, 10 August 2023; 1♂, 26 September 2023; Omalos III, 2♂, 26 May 2023; 1♂, 26 September 2023; 1♂, 2 November 2023; EVROS*: Dadia I, 1♂, 13 August 2012; Dadia II, 1♂, 23 September 2012; LESVOS*: Kratigos, 1♂, 18 April 2004; Parakoila, 1♂, 24 March 2024; Sanatorio Agiasou, 1♂, 3 August 2022; 1♂, 8 October 2022.

**Distribution in Greece:** Known from the provinces of Ioannina, Pieria, and Trikala [26,27]. New for Thrace, North Aegean Isl. and Crete.

**General Distribution:** Albania, Algeria, Andorra, Armenia, Austria, Azerbaijan, Belarus, Belgium, Bulgaria, Croatia, Czech Republic, Denmark, Estonia, Finland, France, Germany, Greece, Gruzia, Hungary, Ireland, Italy, Kazakhstan, Latvia, Lithuania, Moldova, the Netherlands, Norway, Poland, Romania, Russia, Serbia, Slovakia, Spain, Sweden, Switzerland, Turkey, Ukraine, United Kingdom, and Uzbekistan [5,31,32,33,47].

**Remarks:** An euryoecious species with culturophilic tendencies [2]. Larvae are mainly predators of the immature stages of other dipteran species in faeces, but occasionally develop on other substrates, like living snails and insects (acridoid grasshoppers and Lepidoptera), debris in birds’ nests, and the carcasses of small mammals [2,43,55]. It is able to cause urogenital myiasis in humans [2,43].

***Sarcophaga* (*Thyrsocnema*) *kentejana* (Rohdendorf, 1937)**

**Distribution in Greece:** Known from mainland Greece without specific localities [32].

**General Distribution:** Alaska, Austria, Bulgaria, China, Finland, France, Greece, India, Kazakhstan, Mongolia, Norway, Pakistan, Romania, Russia, Sweden, Switzerland, and Ukraine [2,5,31,32,33].

***Sarcophaga* (*Thyrsocnema*) *platariae* (Povolný, 1992)**

**Distribution in Greece:** Known from the province of Thesprotia [5,23,26].

**General Distribution:** Croatia, Greece, and Israel [5,23,31,32,33].

***Sarcophaga* (*Varirosellea*) *uliginosa* Kramer, 1908**

**Distribution in Greece:** Known from mainland Greece without specific localities [31,32,33].

**General Distribution:** Albania, Armenia, Austria, Azerbaijan, Belarus, Bulgaria, Canada, China, Croatia, Czech Republic, Denmark, France, Germany, Greece, Gruzia, Hungary, Italy, Japan, Kazakhstan, Kyrgyzstan, Latvia, Moldova, Mongolia, North Korea, Poland, Romania, Russia, Slovakia, Spain, Tajikistan, Turkey, Ukraine, United Kingdom, and USA [5,31,32,33,47].

**Remarks:** The larvae are considered obligatory predators of the pupae of many species of Lepidoptera [2,43].

**[*Sarcophaga* (*Heteronychia*) *atavina* (Enderlein, 1928)]**

**Distribution in Greece:** Known only from the Dodecanese (Rhodes Isl.) [5,19].

**Remarks:** *S. atavina* was originally described as a species in the subgenus *Pseudodiscachaeta* based on the material collected from the island of Rhodes [19]. After its original description, the species was mentioned again in the first edition of the world catalog for the family, where it was placed as an “Unidentified nominal species-group taxon” of *Heteronychia*, alongside *S. pseudobenaci* [5]. While the validity of the latter taxon as a species has been proved recently [29], *S. atavina* has never been mentioned again in any major work on the subgenus [29,30] and has been excluded from all subsequent catalogs and faunal treatments [31,32]. Due to our inability to locate and examine the type of the species, as well as the lack of new *Sarcophaga* material for examination from the island of Rhodes, we cannot provide any suggestions for the true identity of this taxon.

**[*Sarcophaga* (*Heteronychia*) sp. nr. *violovitshi* (Rohdendorf & Verves, 1979)]**

**Distribution in Greece:** Reported from the province of Attiki [34,35].

**Remarks:** *Sarcophaga violovitshi* is a species of *Heteronychia* endemic to Sakhalin Isl. of Russian Far East that shows strong similarities in cercal and phallic morphology with a few Greek species, like *S. haemorrhoa*, *S. haemorrhoides,* and *S. rondaniana* [5,32,68]. While the species is certainly absent from Greece and the abovementioned record refers to a misidentification, we were unable to properly identify it due to the poor condition of the single male specimen. This individual, deposited and enumerated in the insect collection of the GNHM, was covered in mold and dust, and the phallus was broken off almost from the base and absent from the box in which the specimen was stored. The only available identification character, the cerci, is indeed very similar in shape to those of *S. rondaniana* (which shows the strongest similarities in the morphology of the phallus with *S. violovitshi*), but that alone cannot provide a safe identification.

**[*Sarcophaga* (*Helicophagella*) cf. *novella* Baranov, 1929]**

**Distribution in Greece:** Reported from the provinces of Pieria and Trikala [25,26,27].

**Remarks:** *Sarcophaga novella* is a species of *Helicophagella* belonging to the *S. noverca*-group, the taxonomy of which was perplexed for most of the 20th century until a major revision of the subgenus was carried out in 1997 [42]. Povolný uses the name “*novella*” for at least three different taxa when referring to some of the Greek *Sarcophaga* from the mainland: (1) A larger variety of *S. novercoides* was collected in the lower altitudes around Mt. Olympus [25]. (2) A subspecific name (“*Helicophagella novella* ssp.”) was collected in the provinces of Pieria (Mt. Olympus) and Trikala (Meteora), which possibly refers to the true *S. novella,* and the montane populations of “*S. novercoides*” in the Balkans probably incorporate a number of described and undescribed species of this complex [26]. (3) And, again, it was identified as a subspecies (“*Helicophagella novella* ssp.”) and was collected in the same provinces, which he now identifies as belonging to *Sarcophaga okaliana* (Lehrer, 1975) [27]. Povolný also states, in his treatment of Central European Sarcophagidae, that another morphologically distinct form of the *S. novercoides* complex occurs in the foothills of Mt. Olympus, with *S. novella* being present in the upper altitudes of the Greek mountains and *S. novercoides* probably restricted to the lowlands [2]. Due to our inability to locate and examine the “*novella*” specimens given by Povolný, as well as the lack in this study of newly collected material from the mentioned regions, we defer from placing the above records under *S. novella* or any other species of the *noverca* group. However, it is worth mentioning that although Povolný gave only a single character, namely “the narrow and therefore barely folded membrane of the paraphallus tip” as diagnostic in his description of *S. novercoides* morpha *novella* [25], the provided genital illustrations are almost identical with those of *S. bellae*, a species described after the major revision of the subgenus [42,52] and mentioned for mainland Greece without precise locality data [31,32].

## 4. Discussion

The current checklist of Greek *Sarcophaga* includes 72 species divided in 18 subgenera, a number comparable to those of neighboring countries (Table 3) [5,21,26,27,29,30,31,32,33,43,47]. Among these, two species are considered new for Greece (*S. anaces* and *S. ferox*), one new for Europe (*S. beckiana*), and another verified as present after the examination of fresh material and the recording of precise locality data (*S. bellae*). Within the country, 69 species have been recorded from the mainland (including Peloponnese, Poros Island, and Euboea Island), 25 from the North and East Aegean Islands, 19 from Crete, 17 from Cyclades, 5 from the Dodecanese, and 4 from the Ionian Islands.

The results of the present study suggest possible differences between the *Sarcophaga* spp. communities on the mainland with those of the Aegean Islands. The composition of Sarcophagidae communities in a given area is influenced by both the dominant vegetation type and the degree of human disturbance. Increasing human populations contribute to habitat degradation and ecological fragmentation, leading to the displacement of specialist species and the predominance of a smaller number of generalist species [26,27,69,70,71]. In particular, the long-standing human activity and the harsh climatic conditions of the Aegean Islands have contributed to habitat degradation and fragmentation, resulting in the predominance of xeric communities [72,73,74]. On the contrary, most of the flesh fly studies conducted on mainland Greece were focused in areas with low human activity (coniferous and deciduous forests and alpine habitats) and more favorable climatic conditions [23,24,25,26,27]. As such, orophilic, silvicolous, and strongly synanthropic species are probably sporadically present or even absent in the Aegean Islands, as they are replaced by more xerophilic and culturophilic species [2,23,24,25,26,27].

This study presents the first checklist of the genus *Sarcophaga* in Greece; however, further sampling across different seasons and geographic regions is necessary to elucidate the full scope of biodiversity, distribution patterns, and biogeographic trends. The mainland, the Peloponnese and the mountain ranges and forests of Northern Greece remain insufficiently studied despite the significance of their recognized biodiversity. Similarly, the Ionian Islands and several Aegean Islands (e.g., Euboea, the East Aegean Islands, and the Dodecanese) require further investigation, as their geographic position and diverse habitat types may support additional, rare, or previously unrecorded species.

As necrophagous species commonly found in carrion arthropod assemblages, *Sarcophaga* spp. play a significant role in decomposition processes and are important in forensic entomology [6]. Given the observed differences in species distribution, composition, and environmental factors, further research is needed to evaluate regional variations in their forensic relevance across Greece.

## 5. Conclusions

This study establishes a comprehensive framework for *Sarcophaga* research in Greece, documenting *S. ferox* and *S. anaces* as new records and reporting *S. beckiana* in Europe for the first time. These findings highlight the need for continued faunistic surveys and systematic taxonomic assessments, particularly in underexplored regions, to refine our understanding of flesh fly biodiversity. Beyond the taxonomic value, this research provides ecological insights into habitat preferences, with direct implications for biodiversity conservation and forensic entomology. Given the forensic relevance of *Sarcophaga* spp. in postmortem interval (PMImin) estimation, accurate species identification remains critical for casework applications. Future studies should prioritize targeted collections in unexplored regions of Greece to fully document species diversity. Integrating molecular and morphological approaches will be essential for resolving phylogenetic relationships and refining species identification, advancing both forensic and ecological applications.

## Figures and Tables

**Table 1:** Greek localities providing published records of *Sarcophaga* spp.

Locality No.	Region (County/Island)	Area	Literature
1	Arcadia	Leonidion	[29]
2		Tripolis	[29]
3	Attiki	Akropolis	[25,26,28,29]
4		Anavyssos	[29]
5		Diomedes Botanical Garden	[34,35]
6		Poros Isl.	[5,22,29,30]
7	Crete		[28,29,30,33]
8	Dodecanese	Rhodes Isl.	[29,30]
9	Euboea Isl.		[30]
10	Ilia	Olympia	[30]
11	Ioannina	Pindos Mt.	[2,29]
12		Vikos-Aoos Gorge	[25,26,29,30]
13	Ionian Isl.	Corfu Isl.	[29,30,33]
14	Laconia	Gythion	[29]
15		Mani	[29]
16		Taygetos Mt.	[29]
17	Larissa	Ampelakia	[29]
18		Kokkino Nero	[25]
19		Ossa Mt.	[29]
20	Messinia	Kyparissia	[36]
21	Mt. Parnassus		[5,22,29,30]
22	North Aegean Isl.	Lesvos Isl.	[29]
23	Phthiotis	Thermopylae	[29]
24	Pieria	Katerini	[25]
25		Leptokaria	[25,29]
26		Mt. Olympus	[2,25,26,27,29,30]
27		Platamonas	[12,25,26,27,29,30]
28		Skotina	[5,12,25,26,27,29,30]
29		St. Panteleimon	[25,26,27,29,30]
30		Stavros	[26,27]
31	Preveza	Preveza	[26,27]
32	Thesprotia	Igoumenitsa	[29]
33		Plataria	[5,26]
34	Thessaloniki	Lake Volvi	[30]
35		Stavros	[29]
36		Thessaloniki	[29]
37	Trikala	Kalampaka	[25,29]
38		Meteora	[25,26,27,29]

**Table 2:** List of collection localities of specimens identified in this study.

Locality No.	Region (County/Island)	Area	Coordinates	Ecotype
1	Achaia	Ano Potamia Akratas	38°05′05.9″ N 22°13′08.0″ E	Abandoned mountain settlement
2		Ano Platanos Akratas	38°09′55.9″ N 22°15′14.3″ E	Olive grove
3	Agios Efstratios	Alonitsi	39°31′40.8″ N 25°03′14.4″ E	Phrygana
4	Anafi	Helicodrome	36°21′25.2″ N 25°46′19.2″ E	Phrygana
5		Vagia	36°21′40.3″ N 25°44′46.0″ E	Phrygana
6		Zoodohos Pigi	36°21′29.2″ N 25°49′49.1″ E	Phrygana
7	Andros	Rachi	37°46′08.4″ N 24°58′38.3″ E	Phrygana
8	Antikythera	Antikythera	35°50′41.6″ N 23°18′56.9″ E	Phrygana
9	Anydros	Anydros	36°37′29.2″ N 25°40′59.0″ E	Phrygana
10	Arcadia	Tripoli	37°30′43.2″ N 22°22′37.2″ E	Urban
11	Attiki	Agia Marina	37°59′42.0″ N 23°40′01.2″ E	Urban
12		Agia Varvara	37°59′13.2″ N 23°39′21.6″ E	Urban
13		Agricultural University of Athens	37°58′55.2″ N 23°42′21.6″ E	Urban
14		Althea Beach	37°48′27.0″ N 23°50′56.0″ E	Urban
15		Diomedes Botanical Garden	38°00′25.2″ N 23°38′34.8″ E	Phrygana
16		Ellinikon International Airport	37°53′09.6″ N 23°44′42.0″ E	Urban
17		Ippokrateios Politeia	38°12′50.4″ N 23°48′21.6″ E	Pine Forest
18	Chania	Alikianos	35°26′17.2″ N 23°56′10.2″ E	Citrus and olive orchards
19		Chania	35°30′30.2″ N 24°00′30.0″ E	Urban
20		Hora Sfakion	35°12′07.2″ N 24°08′13.2″ E	Urban
21		Kefali	35°23′18.9″ N 23°54′25.5″ E	Olive groves
22		Lakkoi	35°24′48.4″ N 23°56′32.7″ E	Olive groves
23		Omalos I	35°22′19.1″ N 23°53′39.2″ E	Mountainous
24		Omalos II	35°22′12.2″ N 23°54′24.3″ E	*Junglans regia* and *Cupressus sempervirens* forest
25		Omalos III	35°18′57.6″ N 23°54′53.2″ E	*Zelkova abelicea* forest
26	Chios	Emporios	38°12′14.8″ N 26°01′19.6″ E	Phrygana
27		Manargos	38°27′49.7″ N 25°56′16.1″ E	Sand dunes
28	Delos	Delos	37°24′06.1″ N 25°16′10.6″ E	Phrygana
29	Evros	Dadia I	41°00′05.8″ N 26°15′31.3″ E	No data
30		Dadia II	41°02′23.3″ N 26°10′11.6″ E	No data
31		Dadia III	41°00′11.2″ N 26°09′06.8″ E	No data
32		Dadia IV	40°59′14.6″ N 26°03′29.5″ E	No data
33		Dadia V	40°59′39.5″ N 26°05′35.9″ E	No data
34		Dadia VI	41°00′38.2″ N 26°08′36.6″ E	No data
35		Dadia VII	41°01′22.8″ N 26°10′21.4″ E	No data
36		Dadia VIII	40°59′45.2″ N 26°09′06.8″ E	No data
37		Dadia IX	41°00′10.1″ N 26°05′40.9″ E	No data
38		Dadia X	41°02′42.7″ N 26°15′15.1″ E	No data
39	Folegandros	Agios Georgios	36°39′42.8″ N 24°51′10.8″ E	Phrygana
40	Ios	Agia Theodoti	36°45′13.7″ N 25°19′30.7″ E	Phrygana
41		Kambos	36°45′06.5″ N 25°17′31.6″ E	Phrygana
42	Irakleia	Irakleia	36°50′14.2″ N 25°27′19.9″ E	Urban, Phrygana
43		Livadi	36°50′55.0″ N 25°28′18.5″ E	Phrygana
44		Pigadi Beach	36°49′44.0″ N 25°28′08.4″ E	Phrygana
45	Irakleio	Irakleio	35°20′19.0″ N 25°07′24.6″ E	Urban
46	Karpathos	Avlona	35°46′08.0″ N 27°11′05.6″ E	Phrygana
47	Korinthia	Doxa Lake	37°55′30.0″ N 22°17′27.6″ E	Pine forest
48		Kato Trikala	37°59′53.8″ N 22°28′46.8″ E	Maquis
49		Lechaio	37°56′09.6″ N 22°51′36.0″ E	Urban
50	Lemnos	Agios Athanasios	39°54′18.4″ N 25°04′41.9″ E	Phrygana
51		Atsiki	39°56′35.9″ N 25°11′47.8″ E	Phrygana
52		Moudros I	39°50′22.6″ N 25°18′36.7″ E	Phrygana
53		Moudros II	39°51′51.8″ N 25°17′12.8″ E	Phrygana
54		Plaka-Panagia	39°59′24.0″ N 25°24′48.6″ E	Phrygana
55	Lesvos	Agia Marina	39°03′46.4″ N 26°34′34.7″ E	Urban
56		Alyfanta	39°05′57.9″ N 26°31′14.7″ E	Olive groves
57		Anemotia	39°14′42.0″ N 26°06′28.8″ E	Oak forest
58		Antissa I	39°13′33.0″ N 25°57′39.0″ E	Phrygana
59		Antissa II	39°13′45.0″ N 25°57′05.0″ E	Phrygana
60		Archaia Antissa	39°17′22.6″ N 26°01′09.4″ E	Grassland
61		Castle of Mytilene	39°06′46.8″ N 26°33′39.6″ E	Urban
62		Charamida	39°00′51.0″ N 26°35′26.0″ E	Phrygana
63		Eresos	39°10′19.0″ N 25°55′08.0″ E	Phrygana
64		Karava	39°15′27.4″ N 26°23′37.3″ E	Phrygana
65		Kechrada	39°18′21.6″ N 26°06′46.8″ E	Sand dunes
66		Kratigos	39°02′19.0″ N 26°35′58.0″ E	Phrygana
67		Kremasti Bridge	39°16′15.6″ N 26°15′10.8″ E	Riverbank
68		Latomeio Eresou	39°10′37.0″ N 25°56′45.0″ E	Phrygana
69		Latomeio Pigis	39°09′57.6″ N 26°26′34.8″ E	Olive groves
70		Loutra	39°03′329″ N 26°30′07″ E	Olive groves
71		Loutropoli Thermis	39°10′37.2″ N 26°27′46.8″ E	Olive groves
72		Mistegna	39°12′46.8″ N 26°27′54.0″ E	Urban
73		Moni Ipsilou I	39°13′45.0″ N 25°57′05.0″ E	Phrygana
74		Moni Ipsilou II	39°13′55.2″ N 25°56′09.6″ E	Maquis
75		Moria	39°08′06.0″ N 26°31′51.6″ E	Phrygana
76		Mytilene	39°07′17.0″ N 26°32′54.0″ E	Phrygana
77		Palios	39°19′40.8″ N 26°25′12.0″ E	Rocky beach
78		Pamfila	39°09′25.2″ N 26°31′12.0″ E	Urban
79		Parakoila	39°09′56.2″ N 26°08′20.2″ E	Olive groves
80		Pelopi	39°19′37.2″ N 26°17′27.6″ E	Riverbank
81		Petalidi Beach	39°12′28.8″ N 26°29′16.8″ E	Rocky beach
82		Petra	39°20′13.2″ N 26°10′55.2″ E	Sandy beach
83		Petrified Forest Park “Bali Alonia″	39°12′24.8″ N 25°54′08.3″ E	Phrygana
84		Pirgi Thermis	39°10′33.6″ N 26°30′21.6″ E	Urban, Olive groves, Grasslands
85		Plaka Park	39°12′16.0″ N 25°51′12.0″ E	Phrygana
86		Polichnitos	39°04′37.2″ N 26°11′42.0″ E	Urban
87		Sanatorio Agiasou	39°03′57.6″ N 26°23′24.0″ E	Chestnut forest
88		Sigri I	39°13′38.0″ N 25°51′30.0″ E	Olive groves
89		Sigri II	39°13′51.0″ N 25°50′57.0″ E	Olive groves, Phrygana
90		Skala Eresou	39°08′13.2″ N 25°55′33.6″ E	Sandy beach
91		Skala Pamfilon	39°09′21.6″ N 26°31′48.0″ E	Olive groves, Wetlands
92		Vathylimno Waterfalls	39°12′25.5″ N 26°02′07.9″ E	Riverbank
93		Vatousa	39°14′12.0″ N 26°01′50.0″ E	Oak forest, Phrygana
94		Vigla Pamfilon	39°10′12.0″ N 26°32′09.6″ E	Vineyards, Olive groves
95	Mykonos	Panormos	37°28′45.1″ N 25°21′26.6″ E	Phrygana
96	Naxos	Potamia	37°04′08.7″ N 25°26′35.4″ E	Riverbank
97	Rhodes	Platania	36°14′43.8″ N 28°00′55.8″ E	No Data
98	Santorini	Agios Fanourios	36°28′41.2″ N 25°23′58.2″ E	Phrygana
99		Akrotiri-Faros	36°21′24.8″ N 25°21′41.8″ E	Phrygana
100		Panagia Kalou	36°27′46.8″ N 25°25′25.7″ E	Phrygana
101		Pyrgos	36°22′18.8″ N 25°27′12.2″ E	Phrygana
102	Serifos	Megalo Livadi	37°08′18.6″ N 24°25′53.0″ E	Phrygana
103		Panagia	37°10′45.8″ N 24°29′31.9″ E	Phrygana
104		Sklavogianni	37°09′25.9″ N 24°28′10.9″ E	Phrygana
105	Syros	Ermoupoli	37°26′52.8″ N 24°56′52.8″ E	Urban
106	Tinos	Karya	37°34′11.6″ N 25°10′29.6″ E	Phrygana
107		Laouti	37°32′12.5″ N 25°12′40.7″ E	Phrygana
108		Marlas	37°39′07.9″ N 25°01′51.2″ E	Phrygana

**Table 3:** Comparison of the Greek flesh fly fauna with neighboring countries.

Country	Total Subgenera	Total Species	Endemics
Greece	18	72	1
Albania	14	34	0
Bulgaria	20	85	0
Turkey	23	87	6

## Data Availability

The flesh fly specimens listed in this study are deposited in the personal entomological collections of the authors and the following institutions: Entomological Collection of the Korinthian Museum of Natural History (KMNH), Entomological Collection of the Goulandris Natural History Museum (GNHM), and the Melissotheque of the Aegean and are available from the curators upon request. The datasets generated during this study are available from the corresponding author upon reasonable request.
